# Acute dose-dependent effects of lysergic acid diethylamide in a double-blind placebo-controlled study in healthy subjects

**DOI:** 10.1038/s41386-020-00883-6

**Published:** 2020-10-15

**Authors:** Friederike Holze, Patrick Vizeli, Laura Ley, Felix Müller, Patrick Dolder, Melanie Stocker, Urs Duthaler, Nimmy Varghese, Anne Eckert, Stefan Borgwardt, Matthias E. Liechti

**Affiliations:** 1 Department of Biomedicine and Department of Clinical Research, Clinical Pharmacology and Toxicology, University Hospital Basel, Basel, Switzerland; 2 Department of Pharmaceutical Sciences, University of Basel, Basel, Switzerland; 3 Psychiatric University Hospital, University of Basel, Basel, Switzerland; 4 Transfaculty Research Platform Molecular and Cognitive Neuroscience, University of Basel, Basel, Switzerland

**Keywords:** Drug development, Pharmacology, Human behaviour

## Abstract

Growing interest has been seen in using lysergic acid diethylamide (LSD) in psychiatric research and therapy. However, no modern studies have evaluated subjective and autonomic effects of different and pharmaceutically well-defined doses of LSD. We used a double-blind, randomized, placebo-controlled, crossover design in 16 healthy subjects (eight women, eight men) who underwent six 25 h sessions and received placebo, LSD (25, 50, 100, and 200 µg), and 200 µg LSD 1 h after administration of the serotonin 5-hydroxytryptamine-2A (5-HT_2A_) receptor antagonist ketanserin (40 mg). Test days were separated by at least 10 days. Outcome measures included self-rating scales that evaluated subjective effects, autonomic effects, adverse effects, plasma brain-derived neurotrophic factor levels, and pharmacokinetics up to 24 h. The pharmacokinetic-subjective response relationship was evaluated. LSD showed dose-proportional pharmacokinetics and first-order elimination and dose-dependently induced subjective responses starting at the 25 µg dose. A ceiling effect was observed for good drug effects at 100 µg. The 200 µg dose of LSD induced greater ego dissolution than the 100 µg dose and induced significant anxiety. The average duration of subjective effects increased from 6.7 to 11 h with increasing doses of 25–200 µg. LSD moderately increased blood pressure and heart rate. Ketanserin effectively prevented the response to 200 µg LSD. The LSD dose–response curve showed a ceiling effect for subjective good effects, and ego dissolution and anxiety increased further at a dose above 100 µg. These results may assist with dose finding for future LSD research. The full psychedelic effects of LSD are primarily mediated by serotonin 5-HT_2A_ receptor activation.

## Introduction

Lysergic acid diethylamide (LSD) is a classic serotonergic psychedelic with a broad history of early psychiatric research and recreational use [1, 2]. LSD induces a range of complex alterations of the mind that have been shown to depend on serotonin 5-hydroxytryptamine-2A (5-HT_2A_) receptor stimulation [3–5]. Renewed interest has been seen in using LSD in psychiatric research and to assist psychotherapy [6–9]. However, all recent placebo-controlled high-dose studies of LSD used only single doses [4, 10–13]. No recent data are available on the acute effects of different well-defined psychoactive doses of LSD in humans and within the same study. Therefore, the present study evaluated acute subjective and autonomic effects of LSD across a range of relevant doses in healthy subjects. In contrast to previous studies [10–12], we used pharmaceutically well-defined doses of LSD. We verified LSD content uniformity of the doses and performed a pharmaceutical stability test. We comprehensively determined plasma LSD concentrations over time to document each individual exposure to LSD and defined the pharmacokinetics of LSD across all doses. Psychedelics can induce neuroregeneration [14]. Therefore, we also measured plasma brain-derived neurotrophic factor (BDNF) levels as a possible biomarker for neurogenesis [15]. Furthermore, we evaluated the role of 5-HT_2A_ receptors in the acute effects of a high dose of LSD by administering the 5-HT_2A_ receptor antagonist ketanserin prior to the administration of 200 µg LSD and compared the acute response to the administration of 200 µg LSD alone. Complex acute subjective effects of LSD were determined using validated psychometric instruments that are used internationally and in trials with patients and have been shown to be useful for predicting therapeutic long-term responses [16–19]. We hypothesized that LSD effects would be dose-dependent and blocked by ketanserin. The present LSD dose–response data may be useful for dose finding in future LSD research in healthy subjects and patients.

## Methods and materials

### Study design

The study used a double-blind, placebo-controlled, crossover design with six experimental test sessions to investigate the responses to (i) placebo, (ii) 25 µg LSD, (iii) 50 µg LSD, (iv) 100 µg LSD, (v) 200 µg LSD, and (vi) 200 µg LSD 1 h after ketanserin administration (40 mg). Block randomization was used to counterbalance the different dosing conditions. The washout periods between sessions were at least 10 days. The study was conducted in accordance with the Declaration of Helsinki and International Conference on Harmonization Guidelines in Good Clinical Practice and approved by the Ethics Committee of Northwest Switzerland (EKNZ) and Swiss Federal Office for Public Health. The study was registered at ClinicalTrials.gov (NCT03321136).

### Participants

Sixteen healthy subjects (eight men and eight women; mean age ± SD: 29 ± 6.4 years; range: 25–52 years) were recruited by word of mouth or an advertisement that was posted on the web market platform of the University of Basel. Mean body weight was 69 kg and 78 and 60 kg in male and female participants, respectively. Accordingly, doses/body weight of LSD were 1.3-fold higher in men than women. All of the subjects provided written informed consent and were paid for their participation. Drug administration timing did not consider the menstrual cycle in females for practical reasons. Four women used a hormonal contraceptive and one was menopausal. Exclusion criteria were age <25 years or >65 years, pregnancy (urine pregnancy test at screening and before each test session), personal or family (first-degree relative) history of major psychiatric disorders (assessed by the Semi-structured Clinical Interview for *Diagnostic and Statistical Manual of Mental Disorders*, 4th edition, Axis I disorders by a trained psychiatrist), the use of medications that may interfere with the study medications (e.g., antidepressants, antipsychotics, and sedatives), chronic or acute physical illness (e.g., abnormal physical exam, electrocardiogram, or hematological and chemical blood analyses), tobacco smoking (>10 cigarettes/day), lifetime prevalence of illicit drug use >10 times (except for Δ^9^-tetrahydrocannabinol), illicit drug use within the last 2 months, and illicit drug use during the study period (determined by urine drug tests). The participants were asked to consume no more than 10 standard alcoholic drinks/week and have no more than one drink on the day before the test sessions. Six participants had previously used LSD (1–3 times), eight participants had used methylenedioxymethamphetamine (MDMA) (1–5 times), ten participants had previously used a stimulant, including methylphenidate (four participants, 1–2 times), amphetamine (six participants, 1–3 times), and cocaine (two participants, 1–2 times), and one participant had smoked opium (once). Six participants had never used any illicit drugs with the exception of cannabis. Substance use histories are shown in Table S1 in the Supplementary Methods online. Drug of abuse tests performed once during the screening and once during the study in each subject were negative.

### Study drugs

LSD base (>99% purity; Lipomed AG, Arlesheim, Switzerland) was administered as an oral solution that was produced according to good manufacturing practice in units that contained 100 or 25 µg LSD in 1 ml of 96% ethanol [20]. The exact analytically confirmed LSD content (mean ± SD) of the 25 and 100 µg formulations was 25.7 ± 0.57 µg (*n* = 9 samples) and 98.7 ± 1.6 µg (*n* = 9 samples), respectively. Stability of the formulation for longer than the study period was documented in an identically produced previous batch [20]. One microgram of LSD base that was used in the present study corresponded to 1.25 µg LSD tartrate that was used recreationally and in other studies [9, 21]. Ketanserin was obtained as the marketed drug Ketensin (20 mg, Janssen-Cilag, Leiden, NL) and encapsulated with opaque capsules to ensure blinding. Placebo consisted of identical opaque capsules that were filled with mannitol. A double-dummy method was used. The subjects received two capsules and two solutions in each session: (i) two placebo capsules and placebo/placebo solutions, (ii) two placebo capsules and 25 µg LSD/placebo solutions, (iii) two placebo capsules and 25 µg LSD/25 µg LSD solutions, (iv) two placebo capsules and 100 µg LSD/placebo solutions, (v) two placebo capsules and 100 µg LSD/100 µg LSD solutions, and (vi) two ketanserin capsules and 100 µg LSD/100 µg LSD solutions. At the end of each session and at the end of the study, the participants were asked to retrospectively guess their treatment assignment.

### Study procedures

The study included a screening visit, six 25 h test sessions (each separated by at least 10 days), and an end-of-study visit. The sessions were conducted in a calm hospital room. Only one research subject and one investigator were present during each test session. The test sessions began at 7:45 a.m. A urine sample was taken to verify abstinence from drugs of abuse, and a urine pregnancy test was performed in women. The subjects then underwent baseline measurements. Ketanserin (40 mg) or placebo was administered at 8:00 a.m. LSD or placebo was administered at 9:00 a.m. The outcome measures were repeatedly assessed for 24 h. Standardized lunches and dinners were served at 1:30 p.m. and 6:00 p.m. respectively. The subjects were never alone during the first 16 h after drug administration, and the investigator was in a room next to the subject for up to 24 h. The subjects were sent home the next day at 9:15 a.m.

### Subjective drug effects

Subjective effects were assessed repeatedly using visual analog scales (VASs) [12, 13] 1 h before and 0, 0.5, 1, 1.5, 2, 2.5, 3, 4, 5, 6, 7, 8, 9, 10, 11, 12, 14, 16, and 24 h after drug administration. VASs were assessed each time LSD blood concentrations were measured to allow for PK–PD modeling. The Adjective Mood Rating Scale (AMRS) [22] was used 1 h before and 3, 6, 9, 12, and 24 h after drug administration. The 5 Dimensions of Altered States of Consciousness (5D-ASC) scale [23, 24] was used as the primary outcome measure and was administered 24 h after drug administration to retrospectively rate peak drug effects. Mystical experiences were assessed 24 h after drug administration using the States of Consciousness Questionnaire [25, 26] that includes the 43-item Mystical Effects Questionnaire (MEQ43) [25], 30-item Mystical Effects Questionnaire (MEQ30) [27], and subscales for “aesthetic experience” and negative “nadir” effects. Subjective effects measurements are described in detail in Supplementary Methods online.

### Autonomic and adverse effects

Blood pressure, heart rate, and tympanic body temperature were repeatedly measured [28]. Adverse effects were assessed 1 h before and 12 and 24 h after drug administration using the list of complaints [29].

### Plasma BDNF levels

Plasma BDNF levels were measured at baseline and 6, 12, and 24 h after drug administration using the Biosensis Mature BDNF Rapid ELISA Kit (Thebarton, Australia) [30].

### Plasma LSD concentrations

Blood was collected into lithium heparin tubes. The blood samples were immediately centrifuged, and the plasma was subsequently stored at −80 °C until analysis. Plasma concentrations of LSD and O–H–LSD were determined by ultra-high-performance liquid chromatography tandem mass spectrometry with a lower limit of quantification of 5 pg/ml [20].

### Pharmacokinetic analyses and pharmacokinetic–pharmacodynamic modeling

Pharmacokinetic (PK) parameters were estimated using a one-compartment model with first-order input, first-order elimination, and no lag time in Phoenix WinNonlin 6.4 (Certara, Princeton, NJ, USA) [20]. The predicted concentrations of LSD were then used as an input to the pharmacodynamic (PD) model by treating the PK parameters as fixed and using a sigmoid maximum effect model in the classic PK/PD link model module in WinNonlin [20]. The time to onset, time to maximal effect, time to offset, and effect duration were assessed for the model‐predicted “any drug effect” VAS effect-time plots after LSD administration using a threshold of 10% of the maximum individual response using Phoenix WinNonlin 6.4.

### Data analysis

Peak (*E*_max_ and/or *E*_min_) or peak change from baseline (Δ*E*_max_) values were determined for repeated measures. The values were then analyzed using repeated-measures analysis of variance, with drug as the within-subjects factor, followed by the Tukey post hoc test. The data were analyzed using Statistica 12 software (StatSoft, Tulsa, OK, USA). The criterion for significance was *p* < 0.05. No correction for multiple testing was applied.

## Results

### Subjective drug effects

**Fig. 1:**
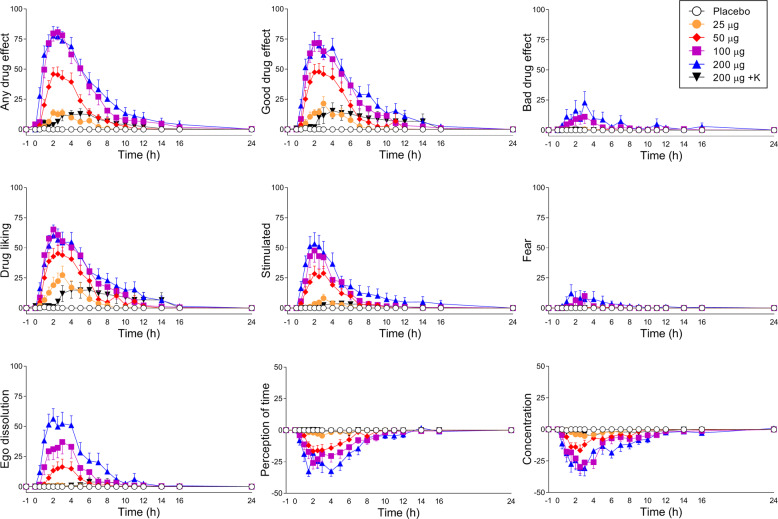
Acute subjective effects of lysergic acid diethylamide (LSD) over time. LSD (25–200 µg) or placebo was administered at *t* = 0 h. Ketanserin (K) or placebo was administered at *t* = −1 h. LSD dose-dependently induced good drug effects, with a maximum effect reached at the 100 µg dose. The 200 µg dose of LSD did not further increase good drug effects or drug liking compared with the 100 µg dose, but it further increased ego dissolution compared with the 100 µg dose. Ketanserin markedly reduced the response to the high 200 µg dose of LSD approximately to the levels of the 25 µg and delayed the remaining small good drug effect and drug liking response compared with LSD alone. The data are expressed as the mean ± SEM percentage of maximally possible scale scores in 16 subjects. The corresponding maximal responses and statistics are shown in Supplementary Table S2.

**Fig. 2:**
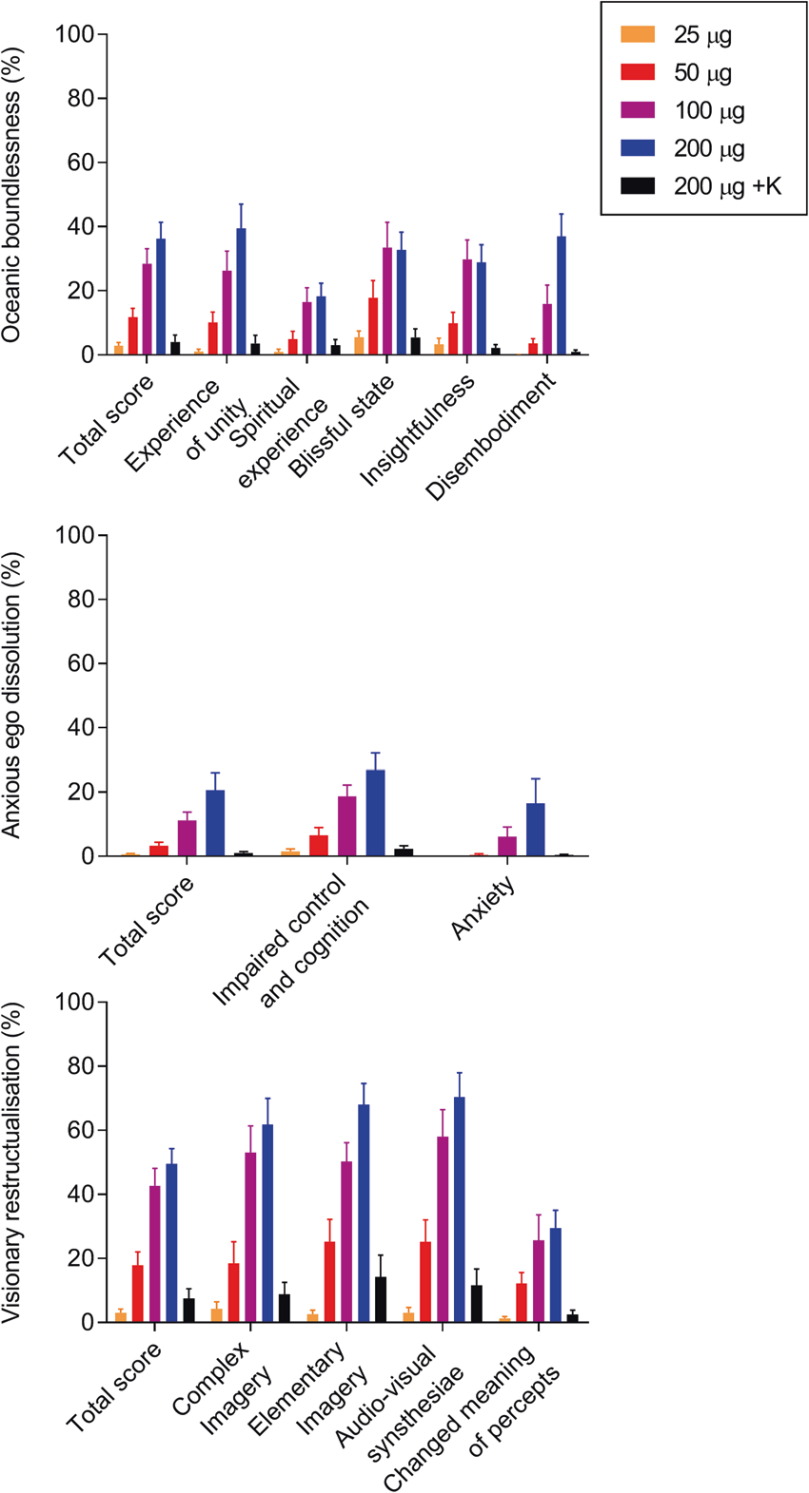
Acute alterations of mind on the 5 Dimensions of Altered States of Consciousness (5D-ASC) Scale. All lysergic acid diethylamide (LSD) doses (25–200 µg) increased “oceanic boundlessness” and “visionary restructuralization.” Only the 50, 100, and 200 µg doses of LSD significantly influenced “anxious ego dissolution.” The dose–response curve showed a ceiling effect for oceanic boundlessness and visionary restructuralization ratings at the 100 µg dose. In contrast, ratings of anxious ego dissolution further increased at the 200 µg dose compared with the 100 µg dose. Additionally, only the 200 µg dose and not the 100 µg dose induced significant anxiety. Ketanserin markedly reduced the response to the highest LSD dose (200 μg) approximately to the level of the 25 µg dose. Placebo scores were too low for visualization. The data are expressed as the mean ± SEM percentage of maximally possible scale scores in 16 subjects. Statistics are shown in Supplementary Table S2.

Subjective effects over time on the VAS and AMRS are shown in Fig. 1 and Supplementary Fig. S1, respectively. The corresponding peak responses and statistics are presented in Supplementary Table S2. Alterations of mind and mystical-type effects are shown in Figs. 2, S2, respectively. Statistics are summarized in Supplementary Table S2.

LSD elicited dose-dependent subjective responses starting at the 25 µg dose, which produced significant “any drug effects” compared with placebo (*p* < 0.05). A ceiling effect was reached at the 100 µg dose of LSD on most scales (particularly positive subjective effects), with typically no significant differences between the 100 and 200 µg doses (Figs. 1, 2,  S2). However, the 200 µg dose produced significantly greater ego dissolution on the VAS (Fig. 1), greater anxious ego dissolution on the 5D-ASC (Fig. 2), and greater subjectively negative nadir effects (Fig. S2) than the 100 µg dose (all *p* < 0.05). Only the 200 µg dose and not the 100 µg dose induced significant anxiety on the 5D-ASC (Fig. 2) and AMRS (Supplementary Fig. S1; both *p* < 0.01). Thus, only ego dissolution and anxiety increased at an LSD dose above 100 µg. Ketanserin significantly (most *p* < 0.001) reduced the subjective response to high-dose LSD approximately to levels that were observed with the 25 µg dose (Figs. 1, 2, S2). Only a small VAS good drug effect in response to LSD was observed after ketanserin administration, which occurred with a temporal delay compared with the effect of LSD alone (Fig. 1). There was no significant difference in the subjective effects of LSD between LSD-experienced and LSD-naïve participants (Fig. S3).

### Autonomic and adverse effects

**Fig. 3:**
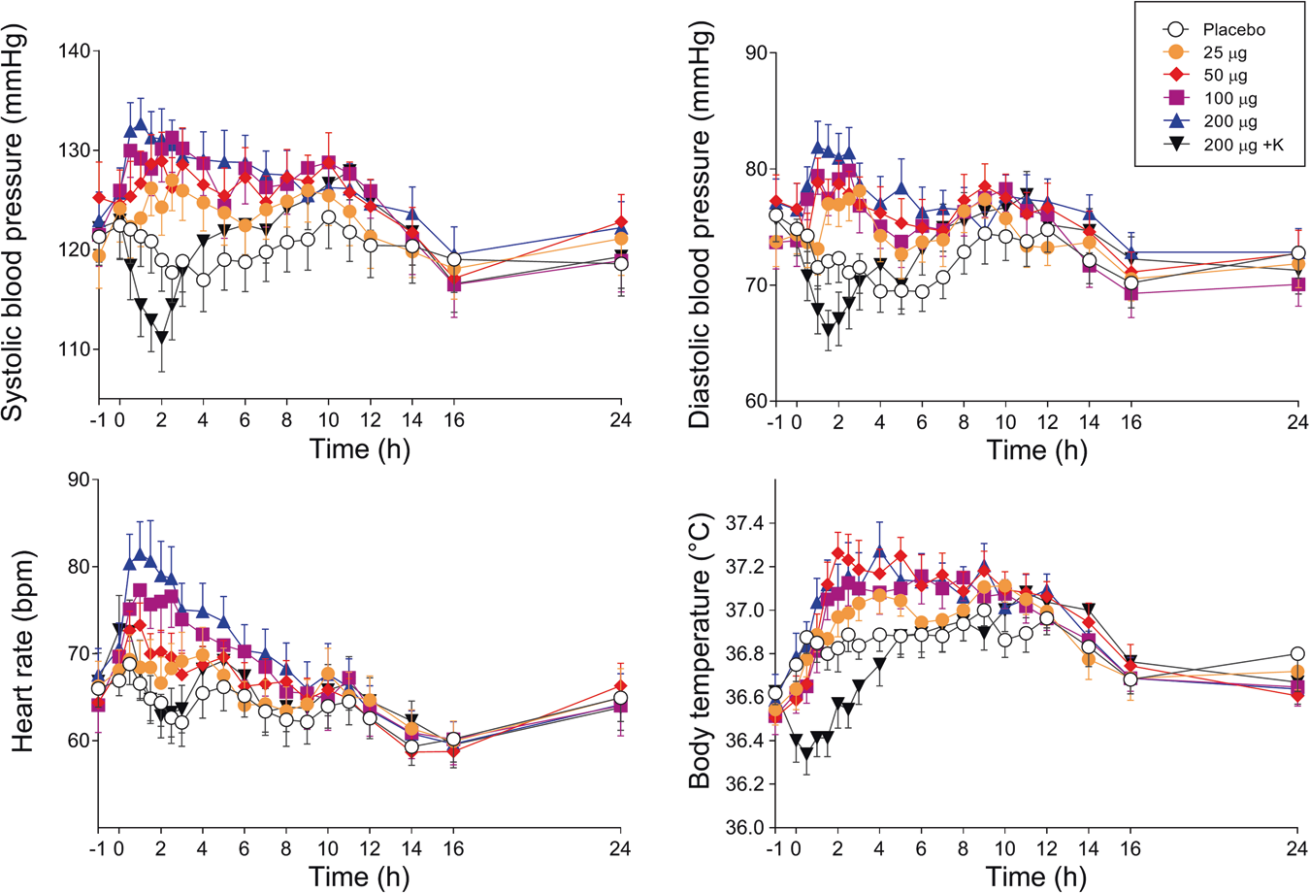
Acute autonomic effects. Doses of 50, 100, and 200 µg lysergic acid diethylamide (LSD) similarly increased systolic blood pressure compared with placebo. The 100 and 200 µg doses similarly increased diastolic blood pressure and heart rate compared with placebo. Ketanserin (K) transiently decreased blood pressure, heart rate, and body temperature, with a delayed increase to the levels that were reached after the administration of LSD alone. LSD (25–200 µg) or placebo was administered at *t* = 0 h. Ketanserin (K) or placebo was administered at *t* = −1 h. The data are expressed as the mean ± SEM in 16 subjects. Maximal effects and statistics are shown in Supplementary Table S2.

Autonomic effects over time and respective peak effects are shown in Fig. 3 and Supplementary Table S2, respectively. Frequently reported adverse effects are presented in Supplementary Table S8. LSD moderately but significantly increased blood pressure at doses of 50 µg or higher and heart rate at 100 and 200 µg (Fig. 3). LSD had no effect on body temperature. LSD at doses of 100 and 200 µg increased the total acute (0–12 h) adverse effects score on the List of Complaints compared with placebo and all other conditions. Ketanserin significantly prevented the LSD-induced heart rate response and transiently reduced the LSD-induced blood pressure response up to 6 h. No severe adverse events were observed.

### Plasma BDNF levels

LSD increased plasma BDNF at the 200 µg dose compared with placebo (Supplementary Table S2, Fig. S4).

### Pharmacokinetics and pharmacokinetic–pharmacodynamic modeling

**Fig. 4:**
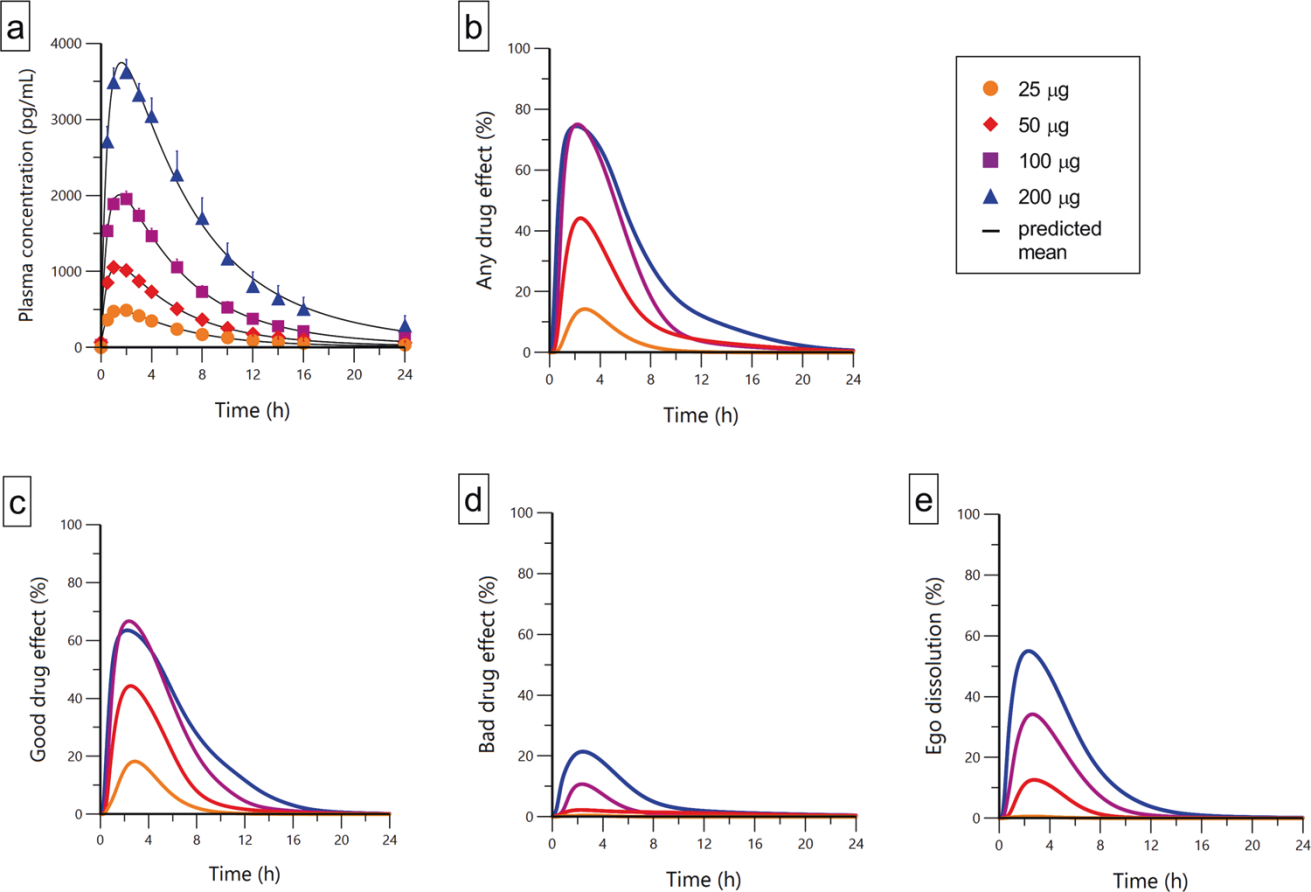
Pharmacokinetics and subjective effects of lysergic acid diethylamide (LSD). **a** Plasma LSD concentration-time curves for 25, 50, 100, and 200 µg doses of LSD. **b–e** LSD effect-time curves for Visual Analog Scale ratings (0–100%) of (**b**) “any drug effect,” (**c**) “good drug effect,” (**d**) “bad drug effect,” and (**e**) “ego dissolution.” LSD administration resulted in dose-proportional increases in plasma concentrations of LSD, but subjective good drug effects reached a ceiling at the 100 μg dose and did not further increase at the 200 µg dose. In contrast, bad drug effects and ego dissolution increased further at the 200 µg dose compared with 100 µg. Therefore, LSD doses higher than 100 µg produced no further increases in good drug effects but more ego dissolution and anxiety. The data are expressed as the mean ± SEM in 16 subjects. LSD was administered at *t* = 0 h. The lines represent the means of the individual pharmacokinetic–pharmacodynamic (subjective effect) model predictions.

Concentrations of LSD and its main metabolite 2-oxo-3-hydroxy LSD (O–H–LSD) could be quantified in all of the subjects, at all doses, and at all time-points. Table 1 shows the PK parameters of LSD. Model-predicted LSD concentrations and effects over time are shown in Fig. 4. Plasma LSD concentrations increased proportionally with increasing doses (Fig. 4). The predicted VAS any drug effects and good drug effects of LSD showed a ceiling effect at the 100 µg dose, and higher bad drug effects and greater ego dissolution were reported at the 200 µg dose compared with 100 µg (Fig. 4). The time to onset, time to maximal effect, time to offset, and effect duration are shown in Table S3. Summarized, the time to onset of the LSD response decreased, and the time to offset increased, resulting in longer effect durations with higher doses of LSD (Fig. 4, Table S3). Ketanserin had no significant effect on the PKs of LSD (Table 1). PK parameters based on non-compartmental analyses are shown in Supplementary Tables S4, S5. Parameters for the PK–PD link model are summarized in Supplementary Table S6. Corresponding individual data is presented in Figs. S5–S24. There were no significant difference in the PKs or effects of LSD between male and female participants.

**Table 1:** Pharmacokinetic parameters for different doses of LSD based on compartmental modeling.

	Dose (µg)		*K*_01_ (1/h)	*λ*_z_ (1/h)	*V*_z_/*F* (L)	*C*_max_ (ng/mL)	*t*_max_ (h)	*t*_1/2_ (h)	AUC_∞_ (ng h/mL)	*CL*/*F* (L/h)
LSD	25	Geometric mean (95% CI)	2.0 (1.3–3.2)	0.19 (0.16–0.24)	38 (30–47)	0.49 (0.41–0.58)	1.2 (0.9–1.7)	3.6 (2.9–4.4)	3.5 (2.7–4.5)	7.2 (5.6–9.3)
		Range	0.40–15.1	0.06–0.35	15–102	0.20–0.71	0.29–2.7	2.0–12	1.1–12	2.0–22
	50	Geometric mean (95% CI)	2.1 (1.5–3.0)	0.19 (0.16–0.23)	35 (31–38)	1.1 (0.99–1.2)	1.2 (0.95–1.6)	3.6 (3.0–4.2)	7.4 (6.2–8.9)	6.7 (5.6–8.0)
		Range	0.43–19.8	0.06–0.44	22–46	0.68–1.6	0.25–2.5	1.6–11	4.2–25	2.0–11.8
	100	Geometric mean (95% CI)	1.8 (1.4–2.4)	0.18 (0.15–0.21)	37 (33–42)	2.0 (1.9–2.2)	1.4 (1.2–1.7)	3.9 (3.2–4.7)	15 (12–18)	6.6 (5.4–8.0)
		Range	0.7–4.5	0.06–0.24	22–51	1.7–2.9	0.74–2.5	2.9–12	11–47	2.1–9.4
	200	Geometric mean (95% CI)	1.6 (1.2–2.1)	0.17 (0.14–0.20)	39 (35–43)	3.9 (3.5–4.3)	1.5 (1.3–1.9)	4.1 (3.4–4.9)	31 (25–38)	6.5 (5.3–8.0)
		Range	0.45–5.03	0.06–0.35	25–67	2.5–6.0	0.70–5.0	2.0–11	18–127	1.6–11
	200 + Ketanserin	Geometric mean (95% CI)	2.3 (1.4–3.8)	0.15 (0.13–0.18)	36 (32–40)	4.4 (4.0–4.8)	1.2 (0.85–1.8)	4.5 (3.8–5.3)	36 (30–43)	5.6 (4.6–6.8)
		Range	0.6–20.0	0.06–0.24	23–47	3.1–6.8	0.25–4.1	0.25–4.1	25–110	1.8–7.9

*AUC*_∞_ area under the plasma concentration–time curve from time zero to infinity, *CL/F* apparent total clearance, *C*_max_ estimated maximum plasma concentration, *t*_1/2_ estimated plasma elimination half-life, *t*_max_ estimated time to reach *C*_max_, *k*_01_ first-order absorption koefficient, *λ*_z_ first-order elimination coefficient, *V*_z_/*F* volume of distribution.

### Blinding

Data on the participants’ retrospective identification of the LSD dose condition are shown in Supplementary Table S7. Generally, the 100 and 200 µg doses were identified as high doses, but these two doses could not be distinguished. The 25 µg dose of LSD was distinguished from placebo and identified correctly or as the 50 µg dose of LSD by most participants. Ketanserin and LSD together were identified correctly or mistaken as a low dose of LSD but never mistaken for a high dose of LSD.

## Discussion

The present study investigated acute effects of LSD using a range of well-defined doses in healthy subjects. Previous recent studies mostly used LSD products that were not developed according to pharmaceutical standards, as discussed elsewhere [20]. Additionally, we determined plasma LSD concentrations as measures of exposure to the substance in the body that are a prerequisite for a valid dose-finding study. We used LSD doses in the psychedelic effect dose range (25–200 µg of LSD base) that were expected to induce full subjective effects of LSD as previously reported by comparable studies that used single-dose levels [4, 10–12]. Plasma LSD concentrations increased proportionally with increasing doses and decreased according to first-order elimination. The PK parameters were consistent with single-dose studies [20, 31]. A preliminary report of a longer terminal elimination half-life of LSD [32] was not confirmed in the present study. We found no sex differences in LSD concentrations or effects consistent with previous studies using no body weight adjustment of LSD doses [20, 31, 32].

LSD dose-dependently increased subjective effects that were largely similar to previous studies that used single-dose levels [10–13, 26]. Importantly, a ceiling effect was reached at higher doses of LSD (>100 µg) with regard to its positive subjective effects, with no difference in good drug effects between the 100 and 200 µg doses. However, the 200 µg dose of LSD produced significantly greater ego dissolution and anxious ego dissolution than the 100 µg dose. Additionally, only the 200 µg dose and not the 100 µg dose of LSD-induced significant anxiety. However, doses above 100 µg may be used if the goal is to induce the experience of ego dissolution or disembodiment. These experiences, however, were produced at doses that also produced more anxiety compared with lower doses. LSD doses of 100 and 200 µg were both subjectively identified as high doses but could not be subjectively distinguished with certainty from each other. Both of these doses can clearly be considered full psychedelic doses and have previously been investigated in healthy subjects [11, 12]. No previous studies directly compared LSD doses of 100 and 200 µg. In contrast to the present findings, we previously reported moderately greater effects of a 200 µg dose of LSD in one study [12] compared with 100 µg in another study [11]. Specifically, 200 µg LSD produced significantly greater total scores on the 5D-ASC scale, including higher ratings of blissful state, insightfulness, and changed meaning of percepts compared with 100 µg [26]. In a previous study, the 200 µg dose of LSD also produced higher ratings of good drug effects, bad drug effects, fear, open, and trust on the VAS compared with 100 µg [11]. There are two explanations for the absence of an LSD dose response for good drug effects in the present study compared with our previous studies. First, the true doses that were used in the previous studies were 60–70 and 150 µg rather than the reported 100 and 200 µg doses because of the use of an unstable formulation with a lower LSD content, as discussed elsewhere [20]. Second, the past comparison was between different subjects and studies [11, 26], whereas the present study used valid within-subject and within-study comparisons. In the present study, we observed a ceiling effect on the dose–response curve. Considering that the previously reported 200 µg dose likely contained only 150 µg of active LSD, additional positive effects may be reached with 150 µg compared with 100 µg. This possibility remains to be tested. One of our recent studies also used an analytically confirmed LSD dose of 100 µg, which produced scores on the VAS and 5D-ASC scale that were nominally higher than those that were reported after 100 µg administration in the present study [13] and more similar to the scores that were reported herein after 200 µg administration. Altogether, the available data support the view that mainly high acute positive effects of LSD can be induced at a 100 µg dose of LSD base. Therefore, we speculate that a dose of 100 µg of LSD may be selected for the treatment of depression or anxiety where higher Oceanic Boundlessness and lower anxiety ratings acutely induced by psychedelics predicted better treatment efficacy [16–19]. The 50 µg dose that was used in the present study also produced substantial positive mood effects and notably only very small and nonsignificant anxious ego dissolution, with no anxiety. Thus, the 50 µg dose may be useful for inducing a moderately intense and predominantly positive psychedelic experience. This low psychedelic dose would likely be a good starting dose to be used in patients with no previous experience with psychedelics or in subjects who are considered to be more sensitive to the effects of psychedelics [33].

In the present study, LSD produced moderate elevations of arterial blood pressure and heart rate starting at the 50 µg dose that were largely similar to the effects of 100 and 200 µg. Similarly, previous studies that used pharmaceutically not well-characterized doses of 100 and 200 µg LSD found no difference in the acute cardiostimulant effects of these doses [11]. A previous study in patients did not observe any increases in blood pressure using a non-confirmed dose of 200 µg of LSD [34]. Methylenedioxymethamphetamine clearly has more pronounced cardiostimulant effects and a less favorable overall physical safety profile than LSD [13, 35]. In contrast, the psychotropic effects of LSD are significantly greater compared with MDMA [13].

In the present study, administration of the 5-HT_2A_ receptor antagonist ketanserin 1 h before LSD administration markedly reduced the subjective response to the 200 µg LSD dose to levels that were similar to the 25 µg dose. Retrospective reports showed that ketanserin and LSD together were identified correctly by the participants or mistaken as a low dose of LSD but never mistaken for a high dose of LSD. The present findings are consistent with a previous study in which ketanserin administration prior to the administration of 100 µg LSD almost completely prevented the acute effects of LSD [4]. These findings support the view that LSD primarily produces its acute psychedelic effects in humans via 5-HT_2A_ receptor activation [3–5, 36], which was also shown for a high and fully psychedelic dose of LSD. Ketanserin also prevented acute adverse effects of LSD and the LSD-induced heart rate response. However, the weak blood pressure-elevating effects of LSD were only transiently prevented by ketanserin and reappeared later during the LSD response. This observation is consistent with the relatively short half-life of ketanserin (i.e., 2 h) during the first 1–9 h following administration [37, 38].

In the present study, 200 µg LSD significantly increased BDNF plasma concentration compared with placebo with a peak at 6 h. Additionally, there were nonsignificant increases in plasma BDNF after lower doses of LSD or after ketanserin with LSD. In previous studies, 100 µg LSD had no effect on BDNF plasma levels [13] up to 5 h while the psychedelic ayahuasca increased BDNF at 2 days. Further, higher BDNF levels were associated with lower depression ratings after administration of ayahuasca [39]. More research is needed to define the time course of the BDNF response and whether there is a link between psychedelics, BDNF, and the antidepressant response [39].

In addition to providing dose–response data on full psychedelic doses of LSD, the present study further characterized the effects of small doses of LSD [9, 40]. The lowest dose that was used in the present study contained 25 µg of LSD base. This dose produced subjective “any drug effects” that were significantly different from placebo and retrospectively identified as LSD by the majority (>85%) of the participants. Very low doses of LSD have typically been referred to as “microdoses.” Psychedelic microdoses have been postulated to have beneficial prolonged effects on mood while producing no or only minimal acute adverse subjective effects [40–43]. Positive long-term effects of psychedelic microdoses remain to be documented [42], and remaining unclear are the LSD doses that actually have no acute subjective effects and thus could be considered microdoses [40]. Very low to low doses of LSD were recently studied in two placebo-controlled trials [9, 21, 44, 45]. One study also provided preliminary PK data [45]. In older healthy volunteers, 5–20 µg of LSD tartrate produced small but significant linear dose-dependent increases in ratings of all of the following: subjective drug effects, vigilance reduction, dizziness, and changes in body feeling [21, 45]. The frequency of adverse effects of LSD at doses up to 20 µg was not different from placebo. The mean plasma *C*_max_ values of LSD (non-compartmental analyses) were 0.44 ng/ml (*n* = 8) after the administration of 20 µg of LSD tartrate [45] and 0.51 ng/ml after the administration of 25 µg of LSD base in the present study, indicating comparable dose-proportional peak concentrations. The previous study included younger healthy subjects and found dose-dependent increases in subjective ratings of “feel drug” and “like drug” on VASs and on the 5D-ASC scale after the administration of 6.5, 13, and 26 µg of LSD tartrate [9]. Notably, a 26 µg dose of LSD tartrate would be lower than the 25 µg dose of LSD base (i.e., 31 µg of LSD tartrate equivalent) that was used in the present study. Nevertheless, the 26 µg dose of LSD tartrate produced significant effects on the 5D-ASC scale compared with placebo and nominally greater ratings on the 5D-ASC subscales than the 25 µg dose that was used in the present study. Unfortunately, no plasma LSD concentration data have been published for the 26 µg dose of LSD tartrate [9]. Therefore, a comparison of drug exposures between this previous study and the present study to further validate the dose comparison is not possible. Altogether, the available data from these controlled studies, including the present study that used very small and small doses of LSD, indicate that the 25 µg dose of LSD is clearly acutely psychoactive in the majority of subjects. Doses in the range of 21–30 µg of LSD base may thus be considered “minidoses” rather than “microdoses.” Doses of LSD base in the 1–20 µg range may be considered “microdoses” but need further study. However, these doses may already elicit small dose-dependent subjective effects, although they are unlikely to relevantly impair cognition or produce adverse effects [9, 21, 44, 45].

Overall, the present dose–response study characterized a range of LSD doses. Based on the available data, the following dosing terminology may be useful for future LSD research: “microdose” (1–20 µg), “minidose” (21–30 µg), and “psychedelic dose” (>30 µg). Within the psychedelic LSD dose range, good effects likely predominate at doses of 30–100 µg (good-effect dose), whereas ego dissolution and anxiety increase at doses above 100 µg (ego-dissolution dose).

The present study has numerous strengths. Four different doses of LSD were used within subjects and compared with placebo under double-blind conditions in a controlled laboratory setting. A ketanserin-LSD condition was also included to elucidate the mechanism of action of LSD and enhance blinding between the different conditions. We also included equal numbers of male and female participants and used internationally established standardized and validated psychometric outcome measures. The doses of LSD were pharmaceutically well-characterized, and plasma LSD concentrations and PK parameters were determined up to 24 h for all doses. Notwithstanding these strengths, the present study also has limitations. The study used a highly controlled setting and included only healthy subjects. Additionally, participants willing to participate in LSD research are likely to have positive expectations and some participants had past substance experiences. Thus, subjects in different environments and patients with psychiatric disorders may respond differently to LSD.

## Conclusion

We characterized the effects of LSD at different doses to support the dosing of LSD for research and LSD-assisted therapy. LSD exhibited dose-proportional PKs and first-order elimination. It produced significant dose-dependent subjective responses starting at the 25 µg dose. A ceiling effect was observed for good drug effects at the 100 µg dose. The 200 µg dose induced more ego dissolution but also more anxiety than the 100 µg dose. These results may assist with dose finding for future LSD research. Ketanserin almost completely prevented the response to the high (200 µg) dose of LSD, thus confirming the critical role of 5-HT_2A_ receptors in mediating psychedelic effects of LSD.

## Funding and disclosure

This work was supported by the Swiss National Science Foundation (grant no. 32003B_185111 to MEL). MEL is a consultant for Mind Medicine, Inc. The other authors declare no competing interests. Knowhow and data associated with this work and owned by the University Hospital Basel were licensed by Mind Medicine, Inc., after study completion. Mind Medicine, Inc. had no role in financing, planning, or conducting the present study or the present publication. Open Access funding provided by Universität Basel (Universitätsbibliothek Basel).

## Supplementary information

Supplementary Material

Table S1

Table S2

Table S3

Table S4

Table S5

Table S6

Table S7

Table S8

Consort Flow Chart
