# Supplementary material for: Acute dose-dependent effects of lysergic acid diethylamide in a double-blind placebo-controlled study in healthy subjects
Source: Neuropsychopharmacology. 2020 Oct 15;46(3):537–44. doi: 10.1038/s41386-020-00883-6 (PMC8027607; doi:10.1038/s41386-020-00883-6)
Supplement: Supplementary file 1 — Supplementary Material [file 41386_2020_883_MOESM1_ESM.docx]

**Supplement**

**Methods**

*Participants*

*Use of medications during the study*

The use of medications that may interfere with the study medications (e.g., antidepressants, antipsychotics, and sedatives) was not allowed. Medications that were not expected to interfere with the study drug were allowed. Eleven participants have used a medication during the time they participated in the study. These medications included analgesics including ibuprofen, diclofenac, acetaminophen, antihistaminics such as fexofenadine, cetirizine, antihistaminic nose drops, nose drops containing phenylephrine or xylometazoline, oral acetylcysteine for common cold, inhalative corticosteroids. None of the drugs were taken on actual study days.

*Subjective drug effects measurements*

*Visual Analog Scales (VASs)*

Subjective effects were assessed repeatedly using visual analog scales (VASs) (1, 2) 1 h before and 0, 0.5, 1, 1.5, 2, 2.5, 3, 4, 5, 6, 7, 8, 9, 10, 11, 12, 14, 16 and 24 h after drug administration. The VASs included “any drug effect,” “good drug effect,” “bad drug effect,” “drug liking,” “stimulated,” “fear”, “ego dissolution,” “concentration,” and “sense of time” (1, 3). The VASs were presented as 100-mm horizontal lines (0-100%), marked from “not at all” on the left to “extremely” on the right. The VAS “perception of time” was bidirectional and marked with “normal” in the middle at 50 mm and “slowed” on the left (0 mm) and “accelerated” (100 mm) on the right. The VAS “concentration” was bidirectional and marked with “normal” in the middle at 50 mm and “not at all” (0 mm) on the left and “extremely” (100 mm) on the right. The primary VAS outcome measures were “any drug effect”, “good drug effect”, “bad drug effect”, and “ego dissolution”. These VAS have been repeatedly used and shown to be sensitive with LSD (1, 2, 4, 5) and were included for the pharmacokinetic-pharmacodynamic modeling as similarly done previously (4, 5). The VAS can relatively rapidly and easily be completed by the participant during the LSD experience and allow to define the drug effect over time. They are sensitive but relatively simple measures. More complex assessments of the LSD state have to be performed primarily at the end of the session and include entire multi-item questionnaires. The VAS “any drug effect” is an overall effect measure to characterize the overall effect intensity and time course. The VAS “good drug effect” is an overall measure of effects subjectively considered positive and interrelated with other measures such as “drug liking”. The VAS “bad drug effect” is an overall measure of any negative effects and related to “fear”. Typically, “bad drug effects” tend to occur at higher doses or plasma concentrations according to previous PK-PD analyses (4, 5). The VAS “ego dissolution” was marked with the sentence: “the boundaries between myself and my surroundings seemed to blur”. This is also an item (nr. 71) of the 5D-ASC which has been used as a simple measure of “ego dissolution” previously (6, 7) and can be used repeatedly as single VAS (1, 4).

VASs were assessed each time LSD blood concentrations were measured to allow for pharmacokinetic-pharmacodynamic modeling.

*Adjective Mood Rating Scale (AMRS)*

The Adjective Mood Rating Scale (AMRS) (8) was used 1 h before and 3, 6, 9, 12, and 24 h after drug administration. The AMRS is a validated 60-item Likert mood rating scale mainly use in Europe and consists of subscales including ratings on “well-being”, “anxiety”, “inactivity”, “extraversion”, “introversion”, and “emotional excitation”. It is suitable for repeated measurements of mood states. The short German EWL60S version was used (8). The completion of the ratings under the effects of psychedelics substances is possible but difficult because it lasts several minutes. The scale was used in paper and pencil version but it may be more suitable to use this measure verbally during states of markedly impaired concentration. The AMRS was included as a secondary supportive measure because it could be considered a better validated measure of mood states and producing more defined ratings than the VAS and to support findings on the VAS (AMRS well-being considered similar to VAS good drug effects; AMRS anxiety considered similar to VAS fear).

*5 Dimensions of Altered States of Consciousness (5D-ASC) scale*

The 5 Dimensions of Altered States of Consciousness (5D-ASC) scale (9, 10) was used as the primary outcome measure and was administered 24 h after drug administration to retrospectively rate peak drug effects. The 5D-ASC scale measures altered states of consciousness and contains 94 items (visual analog scales). The instrument consists of five subscales/dimensions ((9) and 11 lower-order scales (10). The 5D-ASC dimension “Oceanic Boundlessness” (27 items) measures derealization and depersonalization associated with positive emotional states, ranging from heightened mood to euphoric exaltation. The corresponding lower-order scales include “experience of unity,” “spiritual experience,” “blissful state,” and “insightfulness.” The dimension “Anxious Ego Dissolution” (21 items) summarizes ego-disintegration and loss of self-control phenomena associated with anxiety. The corresponding lower-order scales include “disembodiment,” “impaired control of cognition,” and “anxiety.” The dimension “Visionary Restructuralization” (18 items) consists of the lower-order scales “complex imagery,” “elementary imagery,” “audio-visual synesthesia,” and “changed meaning of percepts.” Two additional dimensions describe “Auditory Alterations” (15 items) and “Reduction of Vigilance” (12 items). The total ASC score is the total of the three main dimensions “Oceanic Boundlessness”, “Anxious Ego-Dissolution”, and “Visionary Restructuralization” and can be used as a measure of the overall intensity of the alteration of the mind (7). The scale is well-validated in German (9) and many other languages and widely used to characterize the subjective effects of various psychedelic drugs. In particular, the scale has been used most research groups to psychometrically assess LSD effects (1, 2, 11-14). Furthermore, acute ratings on the 5D-ASC have been used to predict long-term effects of psychedelic treatments in patients (15, 16). Ratings on the 5D-ASC have been shown to closely correlate with ratings on the Mystical Effects Questionnaire (MEQ, see below) (7) which is primarily used by research groups in the US (16).

*Mystical Effects Questionnaire (MEQ30)*

Mystical experiences were assessed 24 h after drug administration using the 100-item States of Consciousness Questionnaire (SOCQ) (7, 17) that includes the 43-item Mystical Effects Questionnaire (MEQ43) (17), 30-item Mystical Effects Questionnaire (MEQ30) (18), and subscales for “aesthetic experience” and negative “nadir” effects. The published German version was used (7). The MEQ has been used in numerous experimental and therapeutic trials with psilocybin (16, 17, 19-25). The MEQ items provide scale scores for each of seven domains of mystical experiences: internal unity, external unity, sacredness, noetic quality (as real as or more real than everyday reality), deeply felt positive mood, transcendence of time and space, and ineffability/paradoxicality (difficulty describing the experience in words). The total of all scale scores was used as an overall measure of the mystical-type experience. We also derived the four scale scores of the newly validated revised 30-item MEQ: mystical, positive mood, transcendence of time and space, and ineffability (18). A complete mystical experience was defined as scores ≥ 60% on all MEQ30 factors (18). While we prefer the German 5D-ASC scale, the German version of the MEQ was also included to facilitate comparison of our findings with those from research using the MEQ (mainly US). Additionally, some aspects of the LSD experience may be better captures with this scale. For the scale validation see (18). For an analysis of the interrelation of the two measures with regards to responses to LSD see (7). For the German translation of the MEQ30 see online supplement of (7).

**
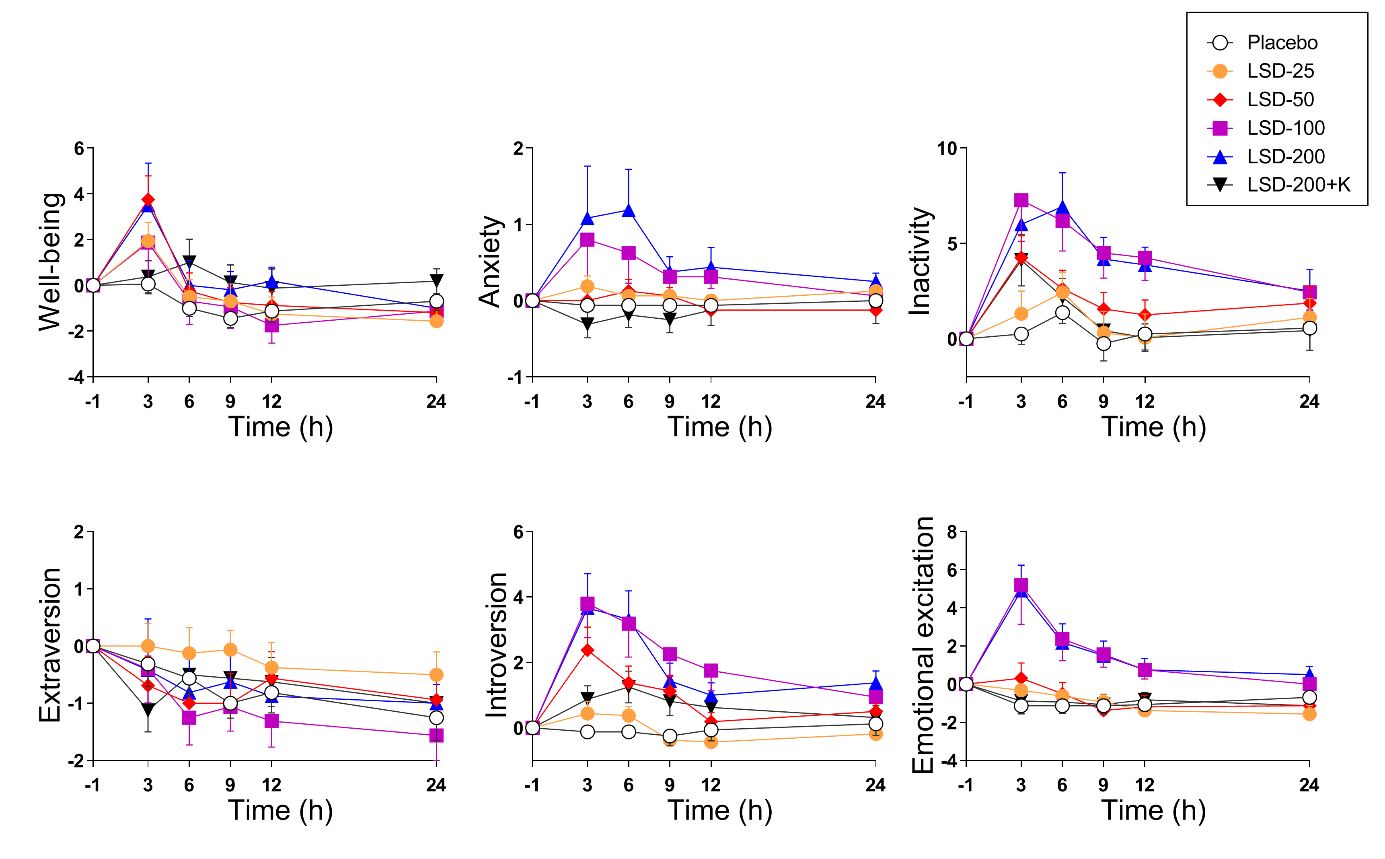
Results**

**Figure S1.** Subjective effects over time on the Adjective Mood Rating Scale (AMRS). The data are expressed as mean ± SEM changes from baseline. Higher doses of lysergic acid diethylamide (LSD; 100 and 200 µg) increased ratings of introversion, inactivity, and emotional excitation compared with placebo. Only the 200 µg dose of LSD significantly increased ratings of anxiety compared with placebo. Only the 50 µg dose of LSD significantly increased ratings of well-being compared with placebo. LSD had no effect on ratings of extraversion. LSD (25-200 µg) or placebo was administered at t = 0 h. Ketanserin (K) or placebo was administered at t = -1 h. The corresponding maximal effects and statistics are shown in Supplementary Table S2.


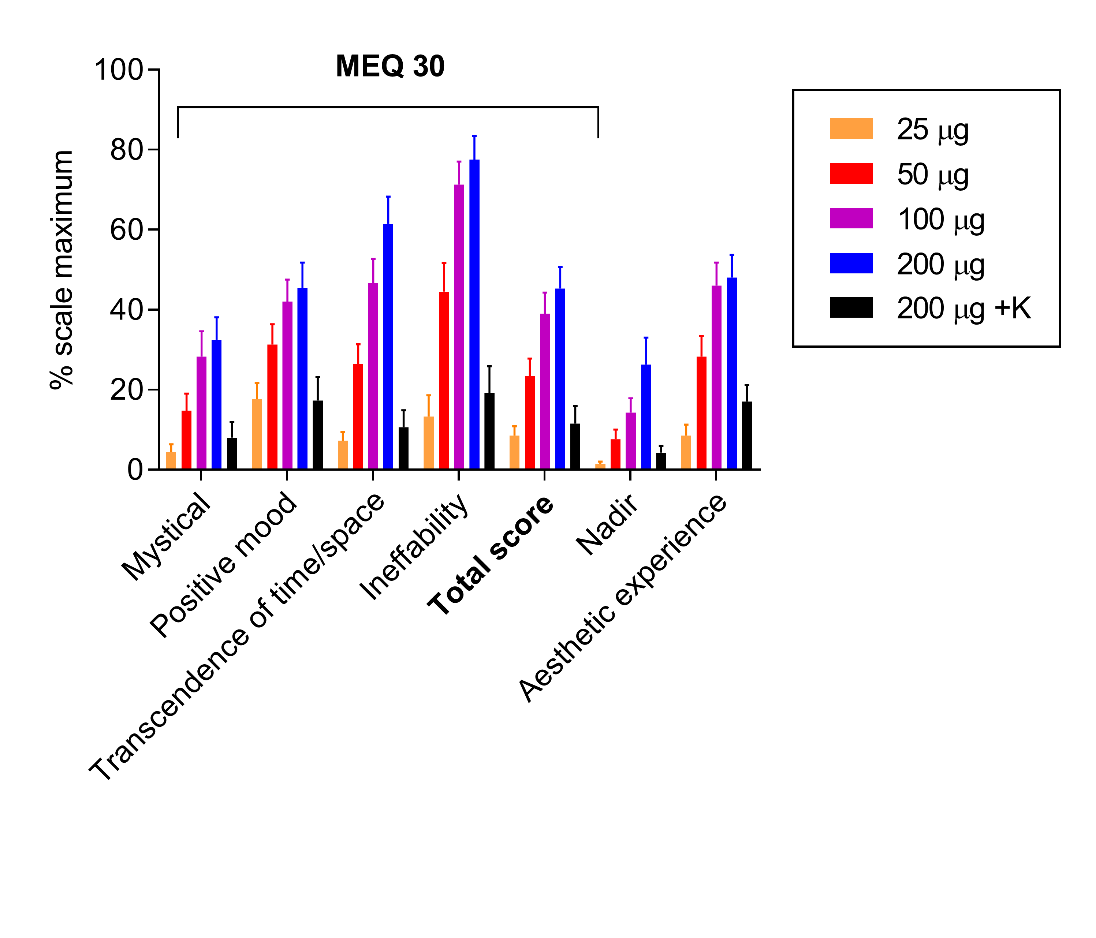


**Figure S2**. Acute mystical-type experiences on the Mystical Effects Questionnaire (MEQ). Lysergic acid diethylamide (LSD) dose-dependently increased MEQ scale ratings, with a trend toward a ceiling effect at the 100 µg dose. Only “nadir” scores, reflecting negative experiences, increased at the 200 µg dose compared with the 100 µg dose. Placebo scores were too low for visualization. Ketanserin markedly reduced the response to the highest LSD dose (200 μg) approximately to the level of the 25 µg dose. The data are expressed as the mean ± SEM percentage of maximally possible scale scores in 16 subjects. Statistics are shown in Supplementary Table S2.


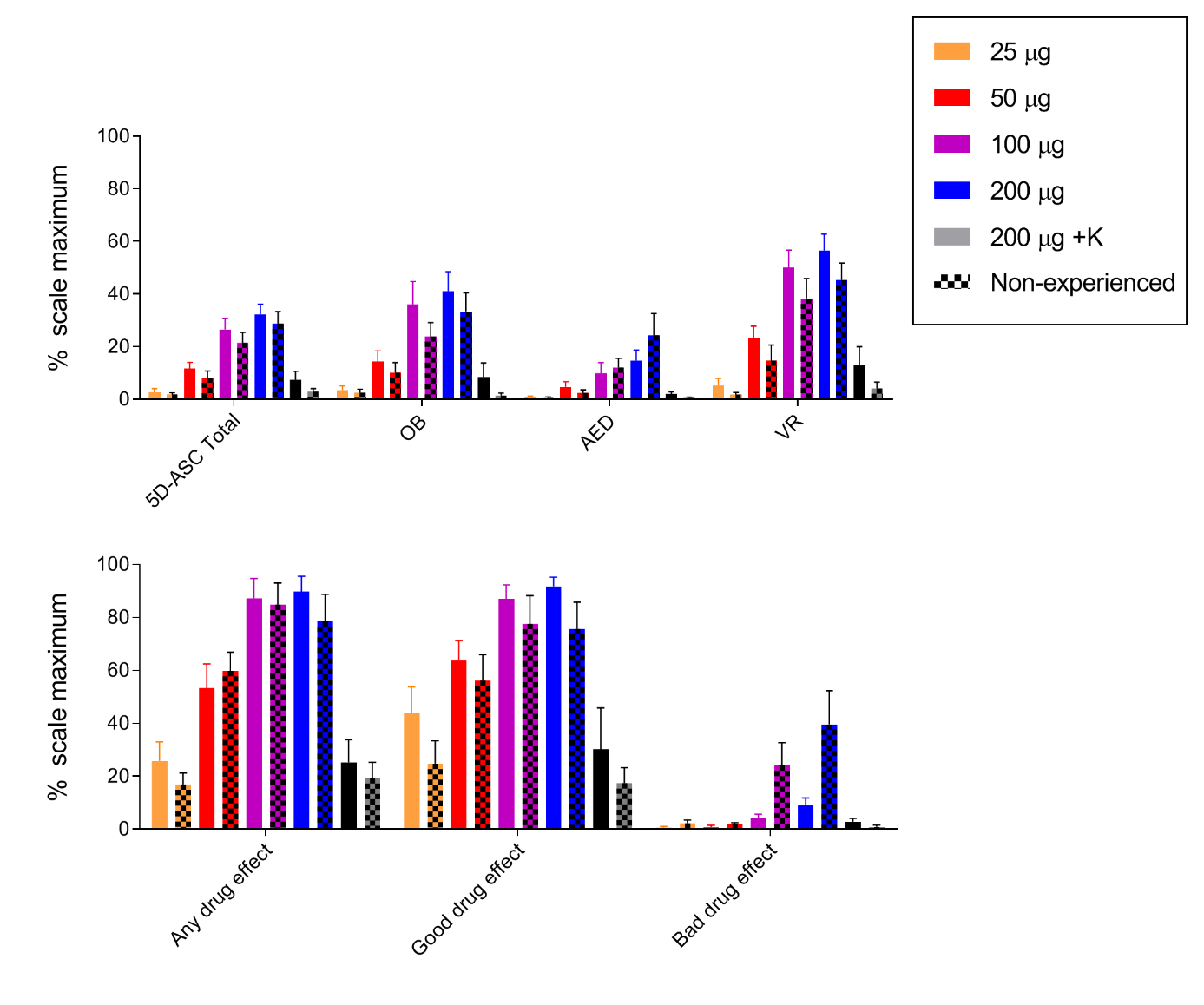


**Figure S3**. Subjective responses on the main 5D-ASC and VAS (E_max_) scales in LSD-experienced (n=6) and LSD-naïve participants (n=10). The data are expressed as mean ± SEM. There was no statistically significant difference between both groups.


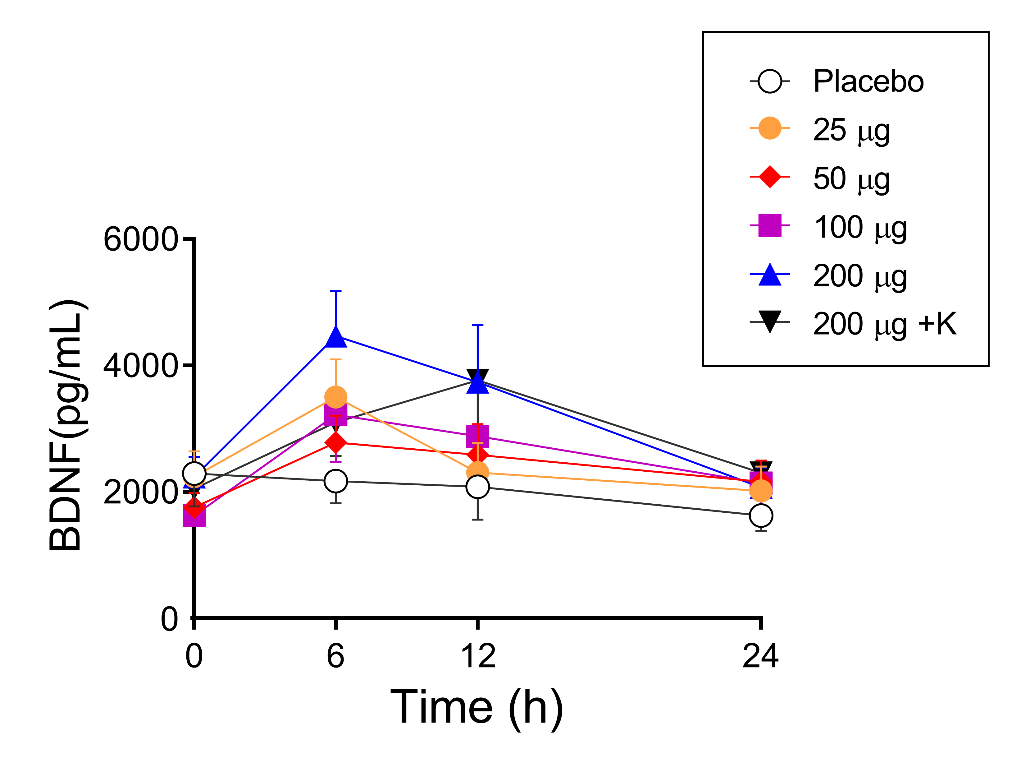


**Figure S4.** Plasma concentrations of Brain-Derived Neurotrophic Factor (BDNF). The data are expressed as mean ± SEM. LSD (25-200 µg) or placebo was administered at t = 0 h. Ketanserin (K) or placebo was administered at t = -1 h. The corresponding maximal effects and statistics are shown in Supplementary Table S2.

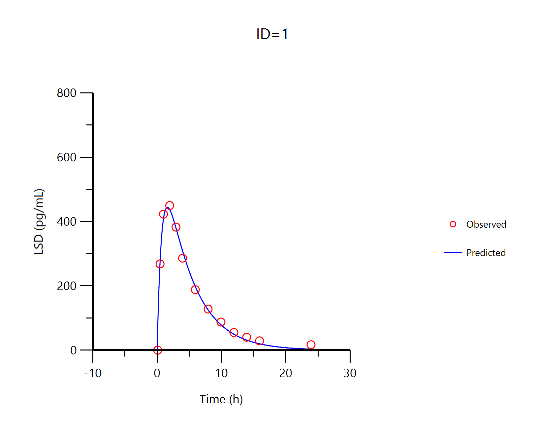

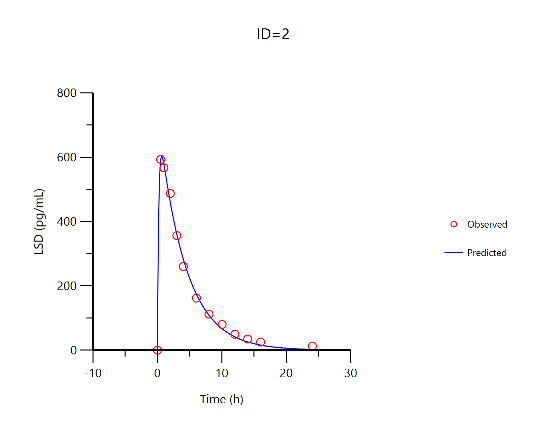

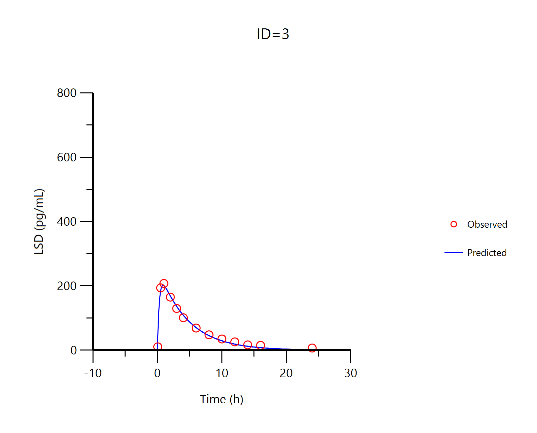

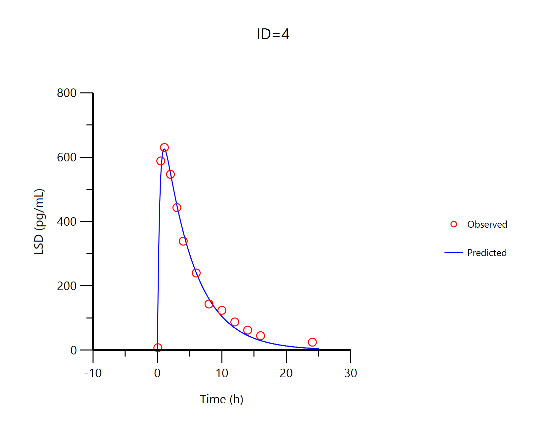

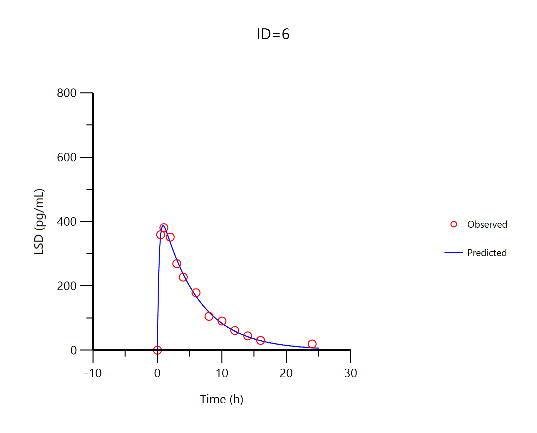

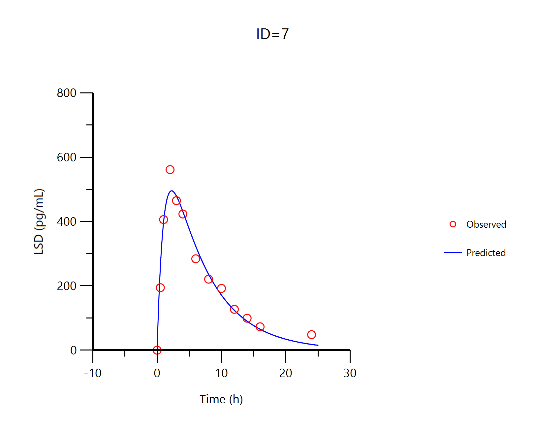

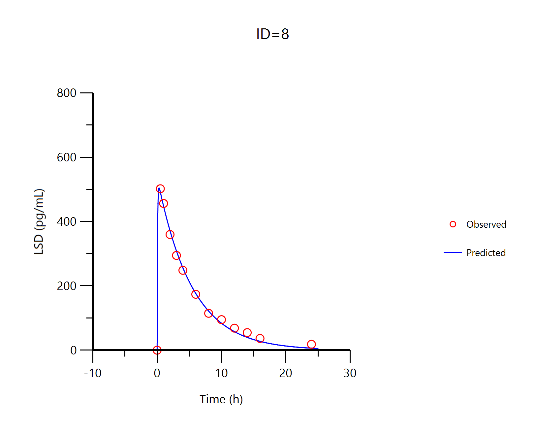

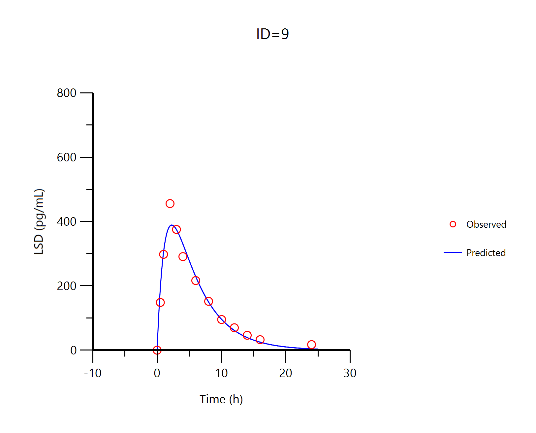

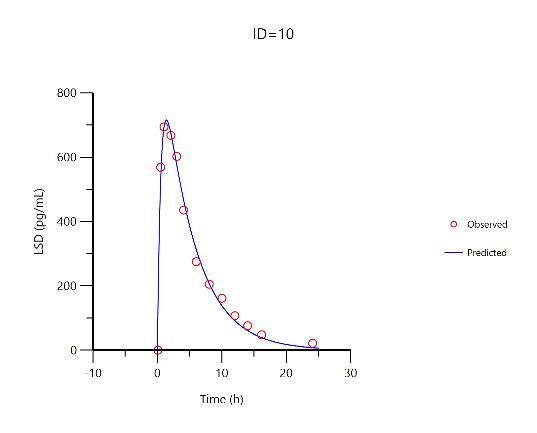

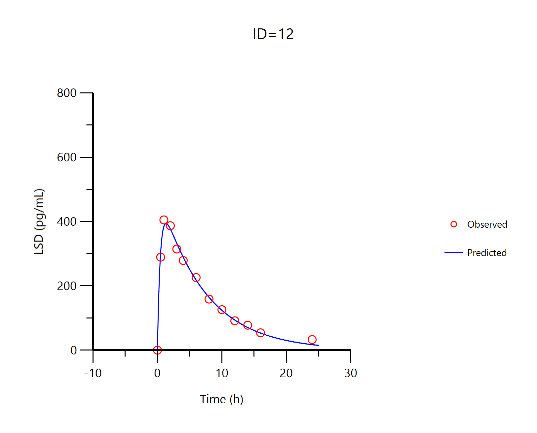

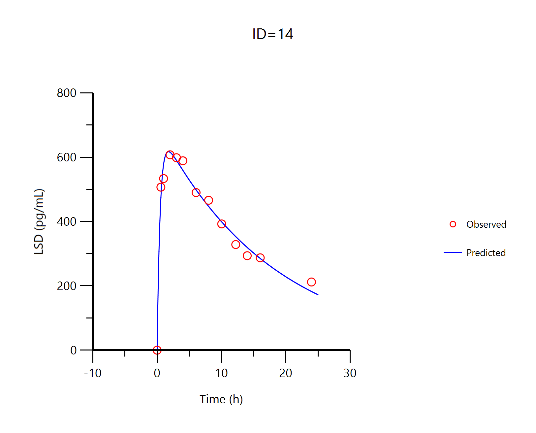

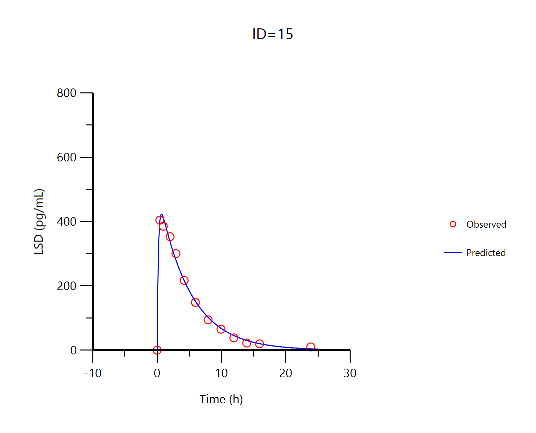

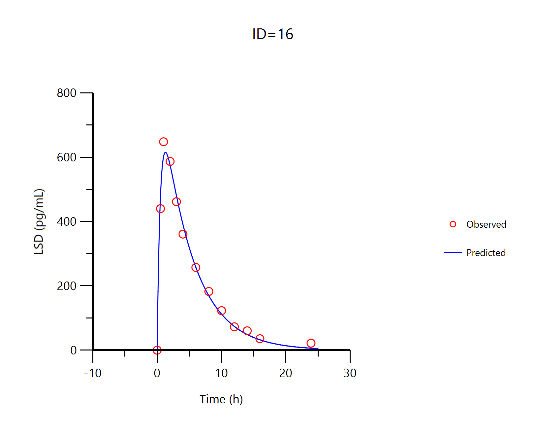

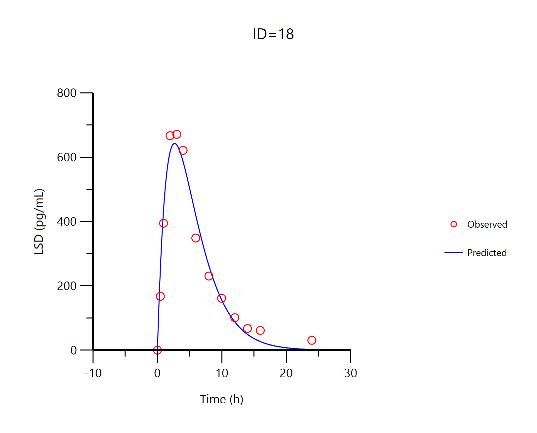

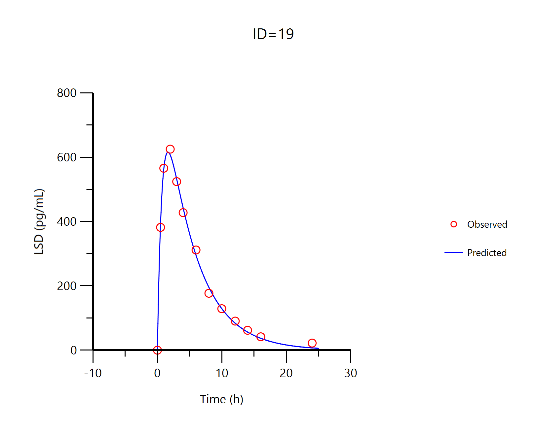

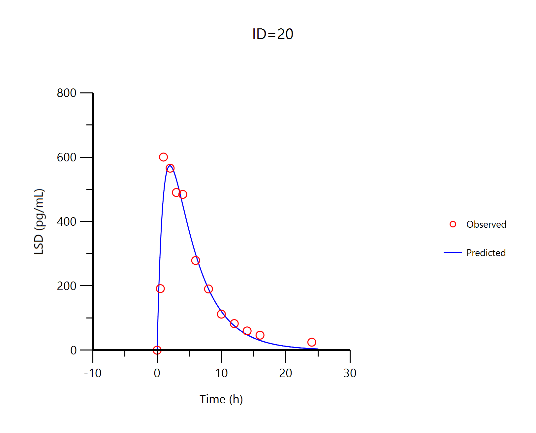


**Figure S5.** LSD plasma concentration-time curves. LSD was orally administered as a solution in ethanol at a dose of 25 µg at t = 0. The data represent individual observed LSD plasma concentrations as measured at the different time points (○) and the LSD concentrations predicted by the one-compartment pharmacokinetic model (blue lines).


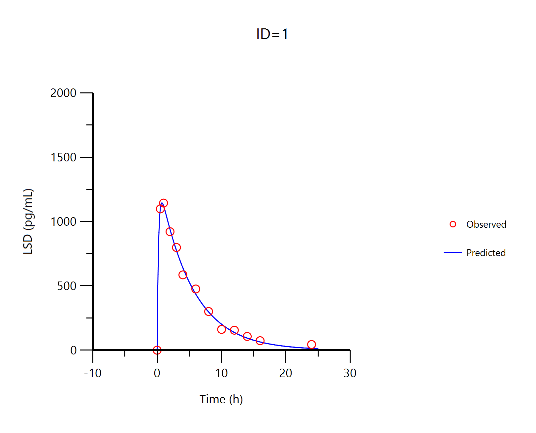

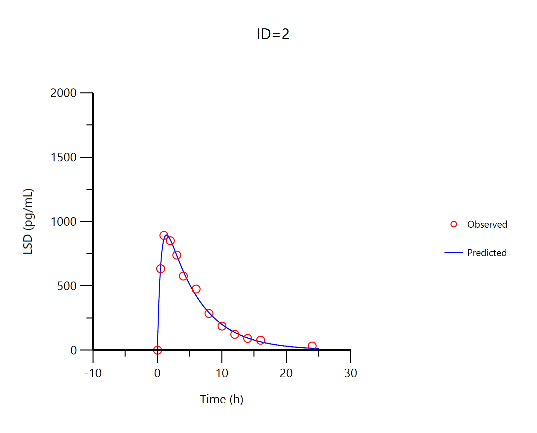

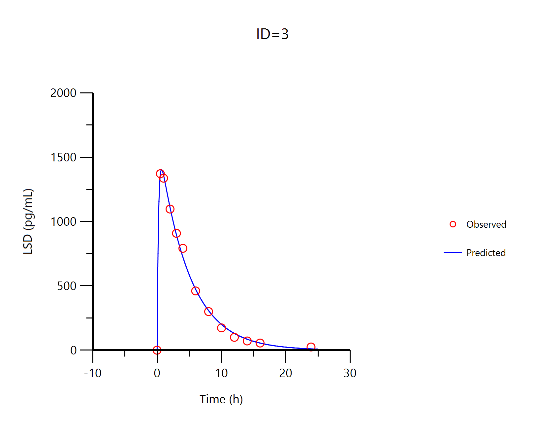

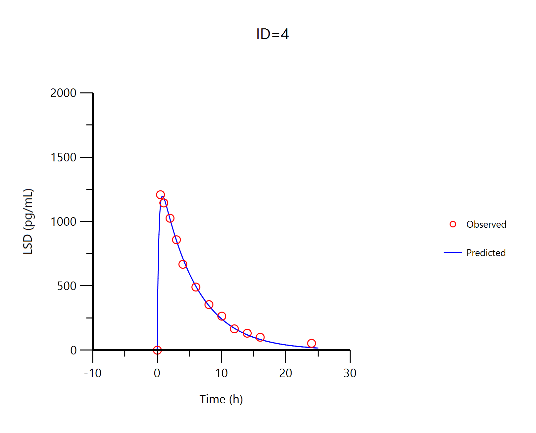

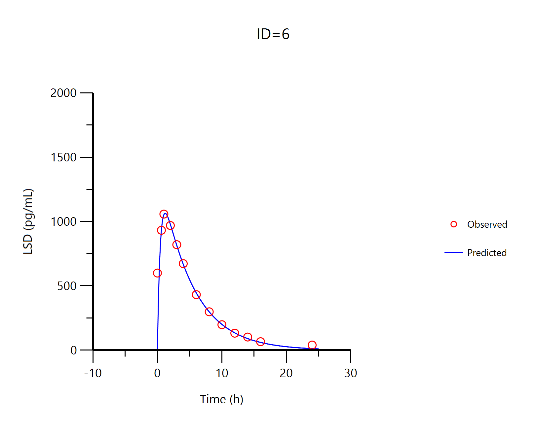

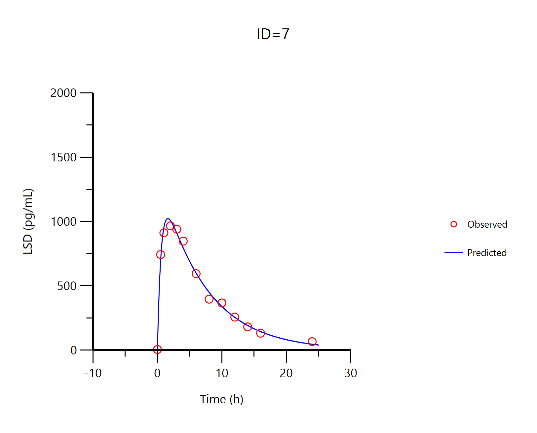

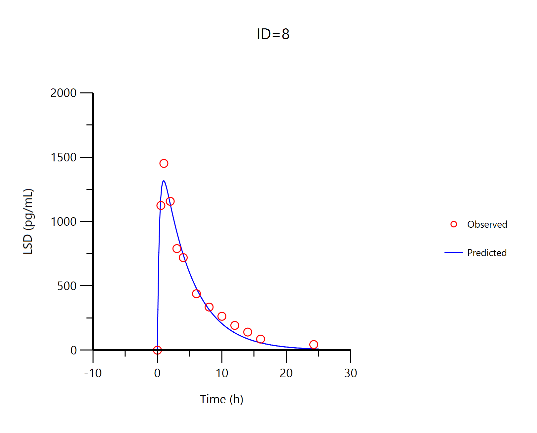

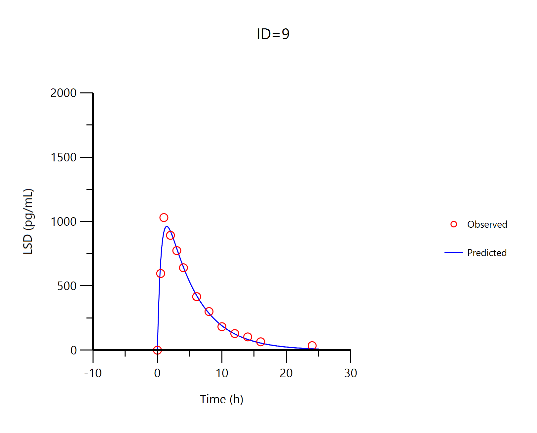

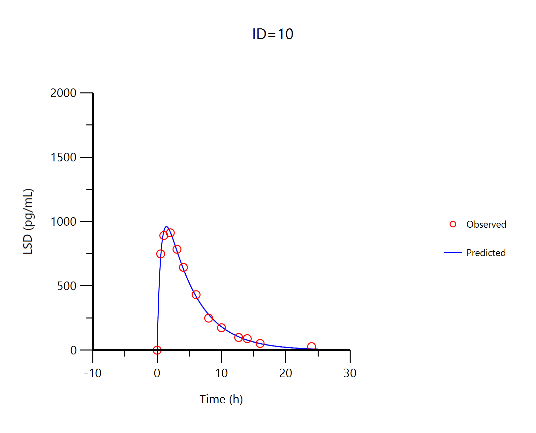

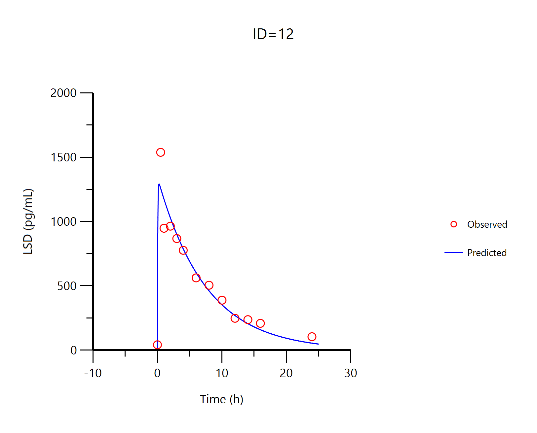

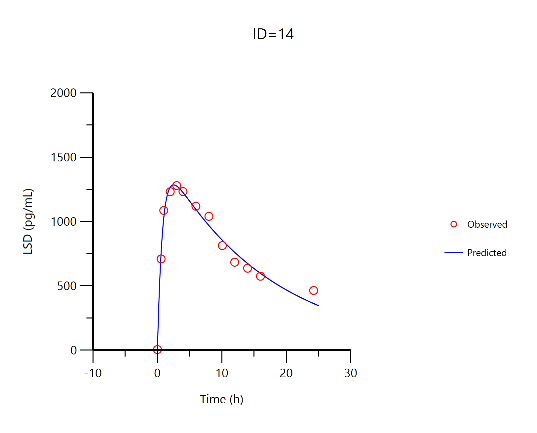

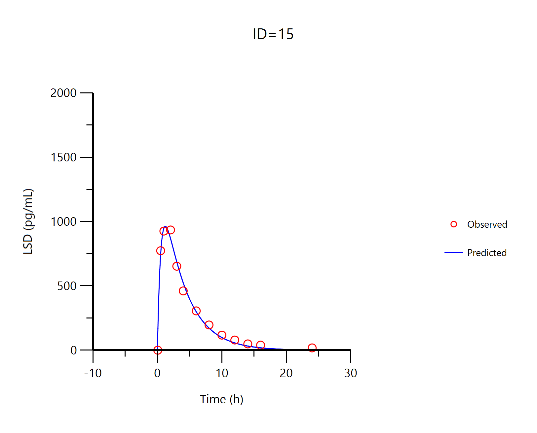

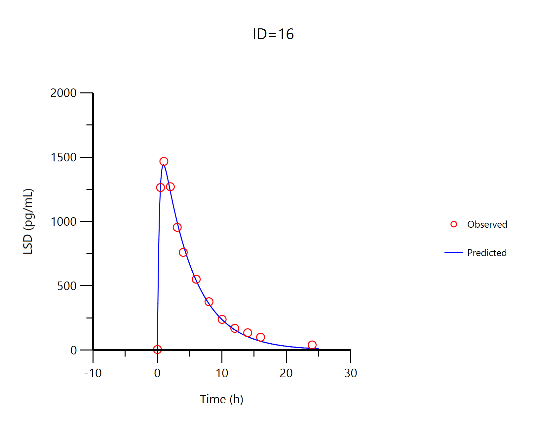

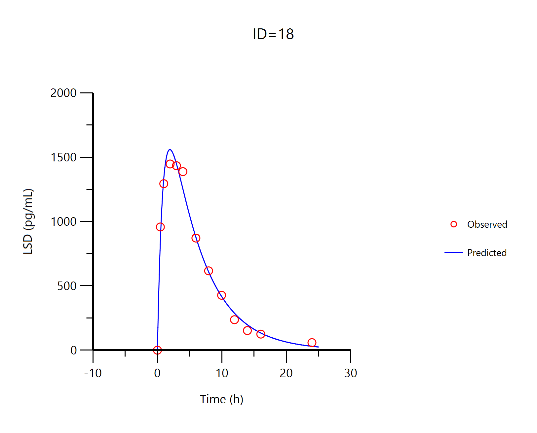

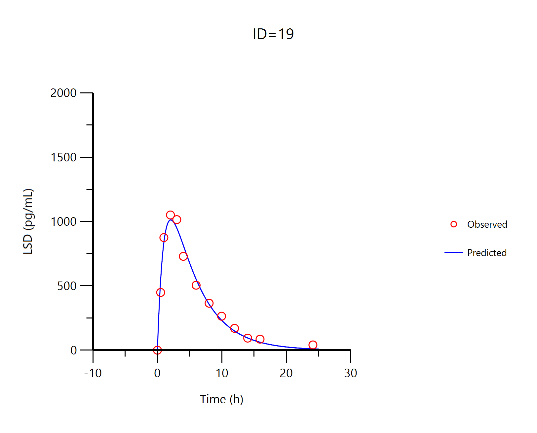

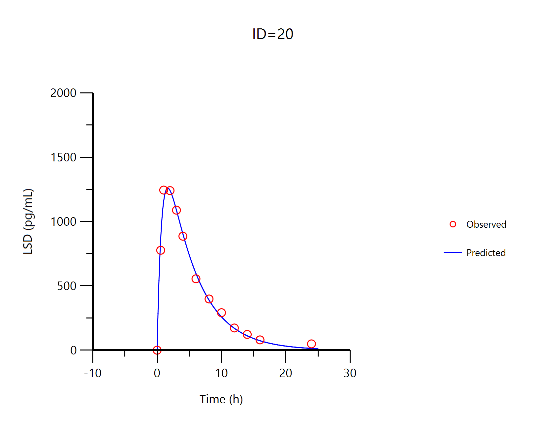


**Figure S6.** LSD plasma concentration-time curves. LSD was orally administered as a solution in ethanol at a dose of 50 µg at t = 0. The data represent individual observed LSD plasma concentrations as measured at the different time points (○) and the LSD concentrations predicted by the one-compartment pharmacokinetic model (blue lines).


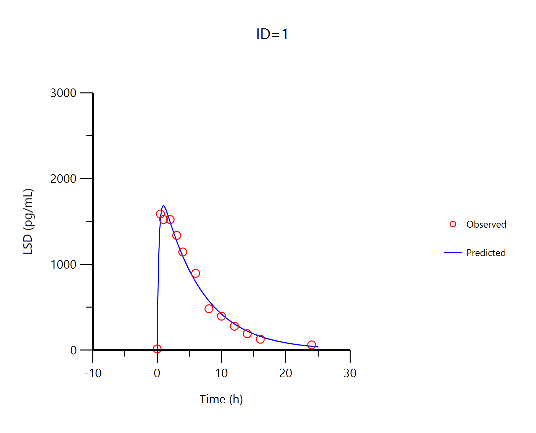

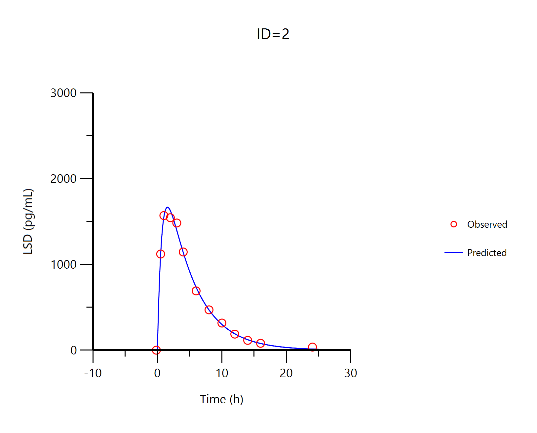

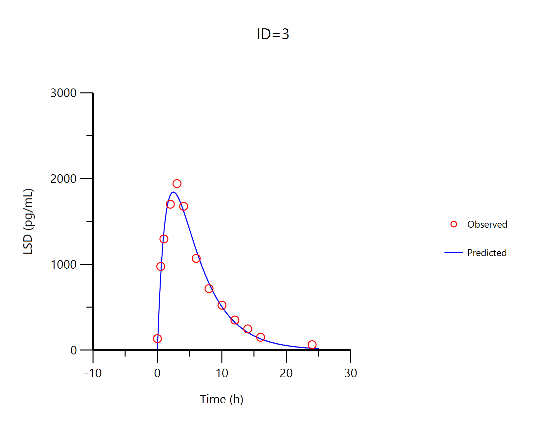

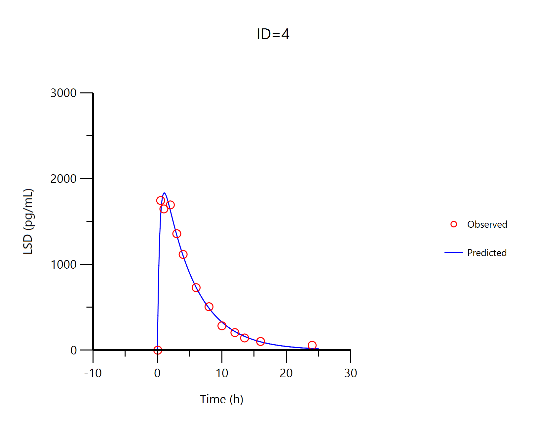

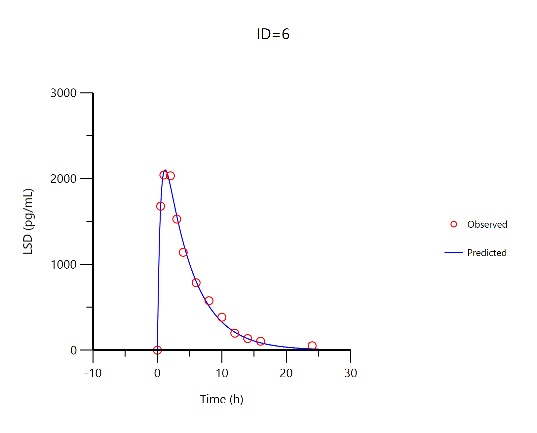

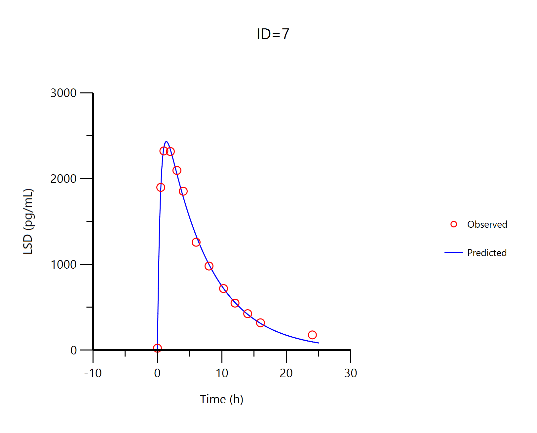

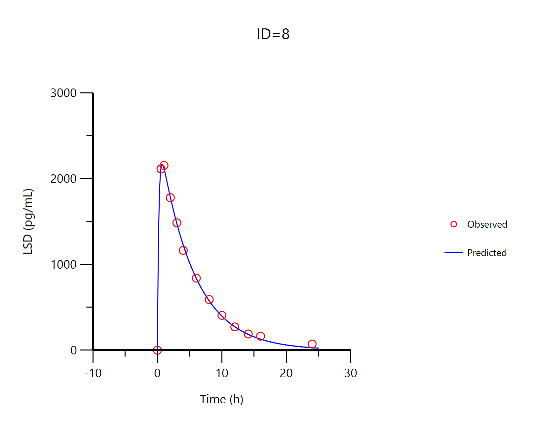

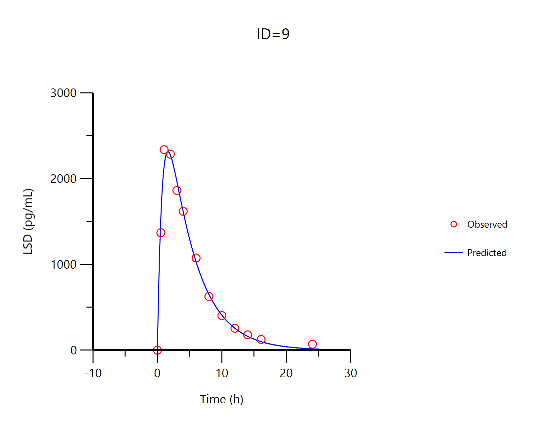

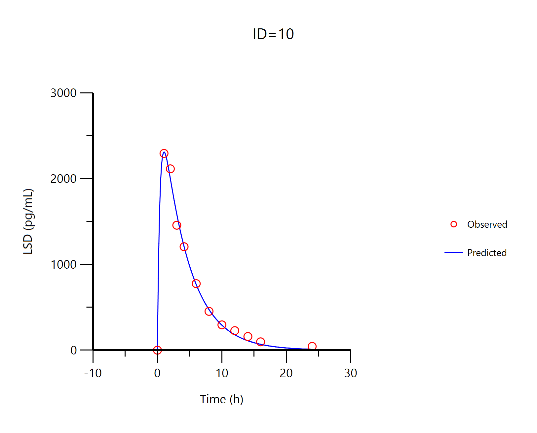

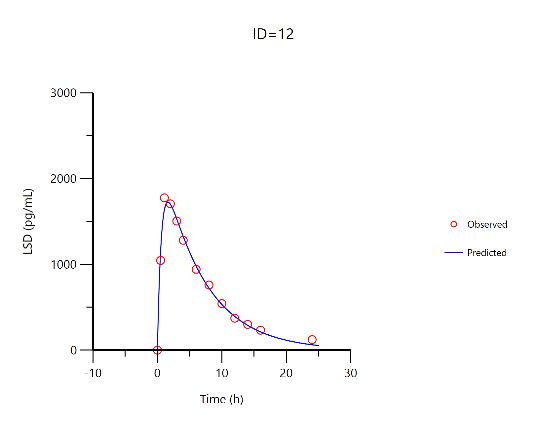

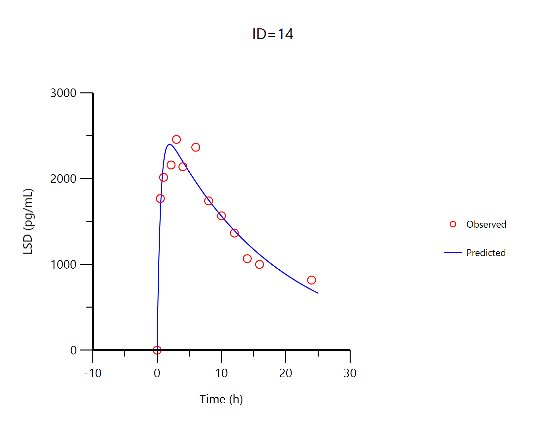

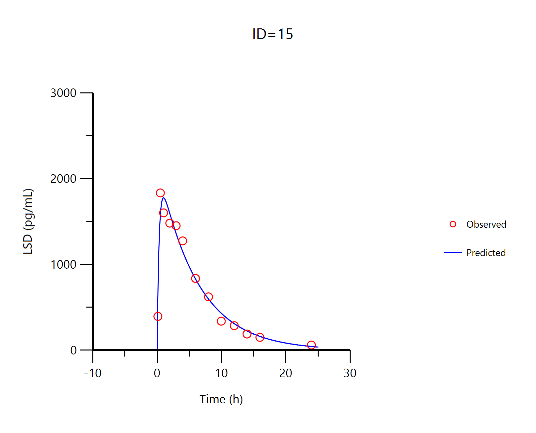

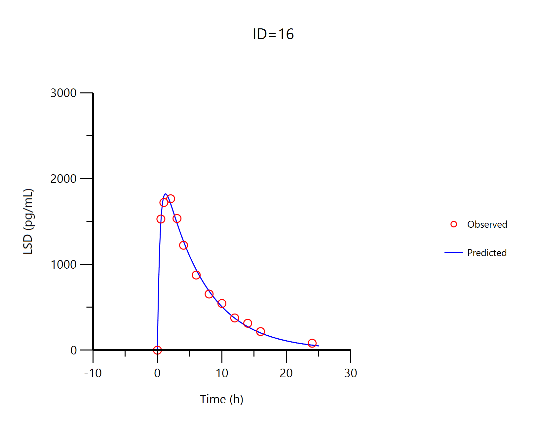

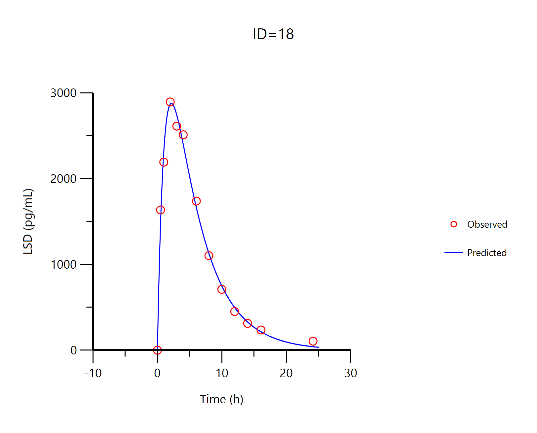

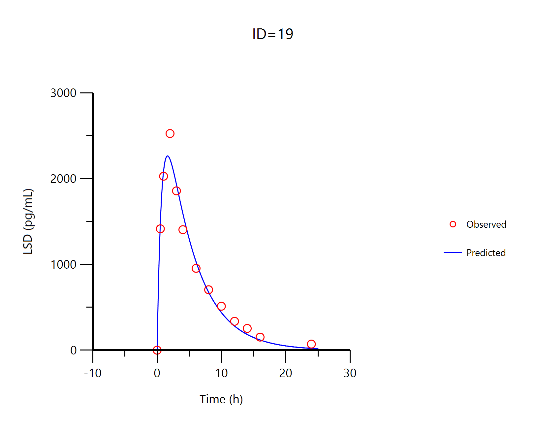

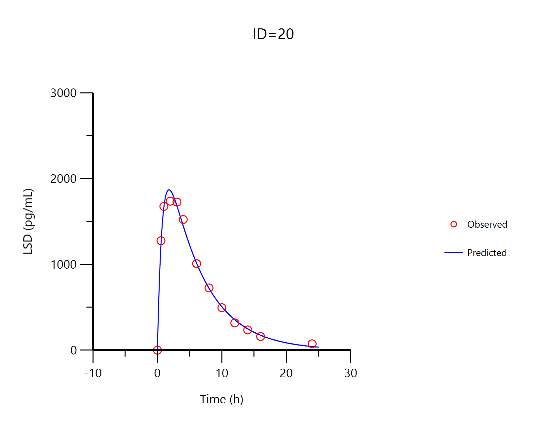


**Figure S7.** LSD plasma concentration-time curves. LSD was orally administered as a solution in ethanol at a dose of 100 µg at t = 0. The data represent individual observed LSD plasma concentrations as measured at the different time points (○) and the LSD concentrations predicted by the one-compartment pharmacokinetic model (blue lines).


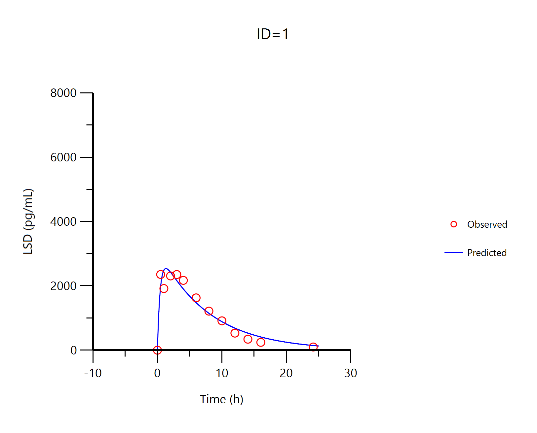

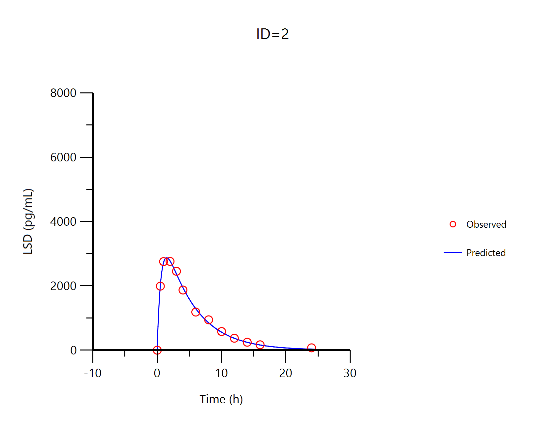

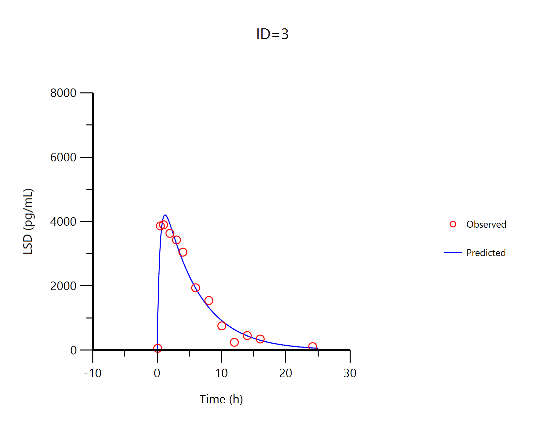

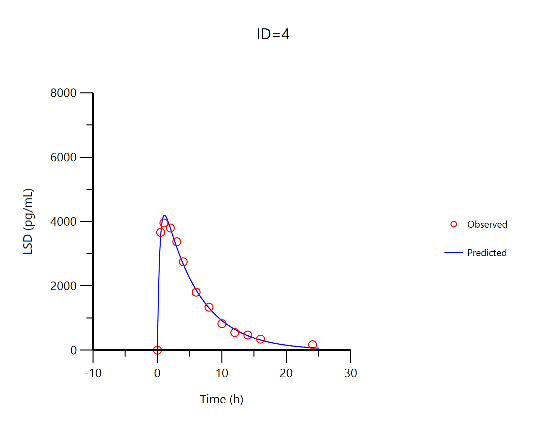

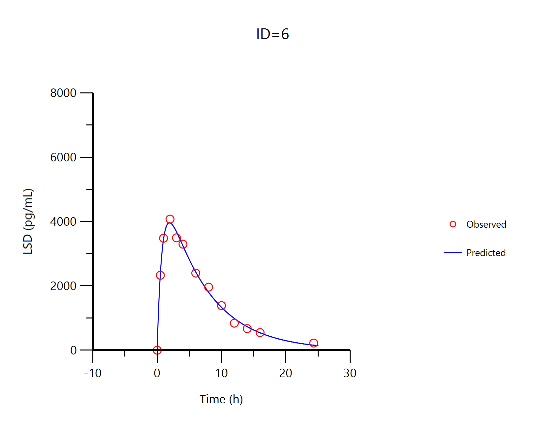

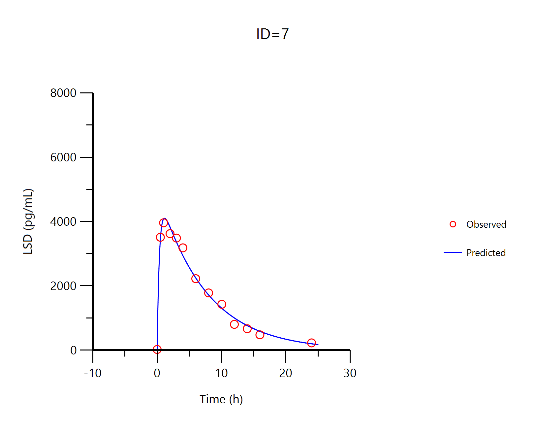

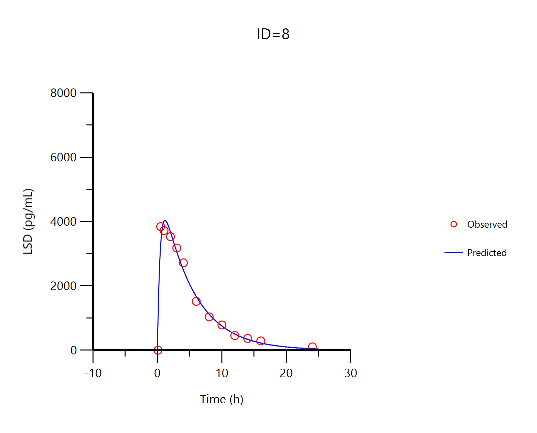

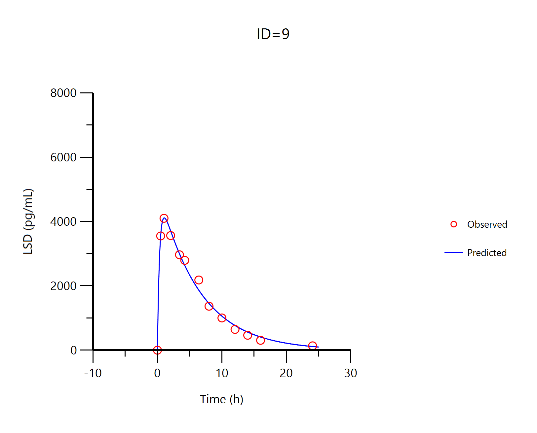

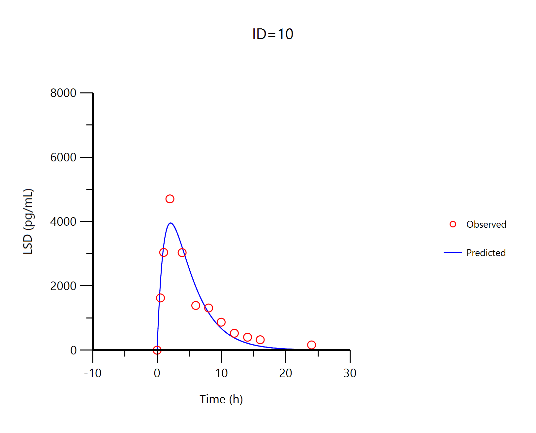

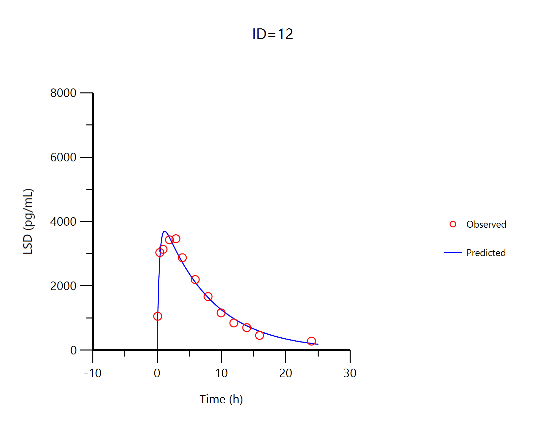

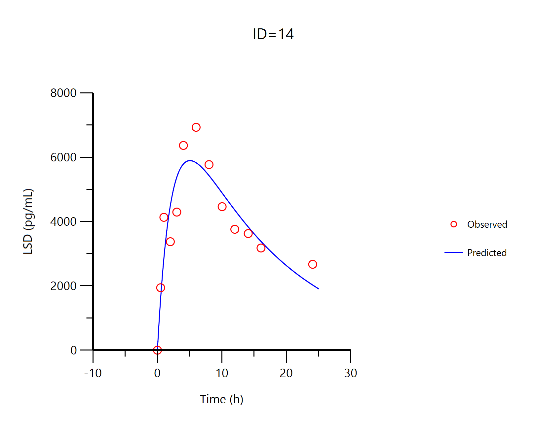

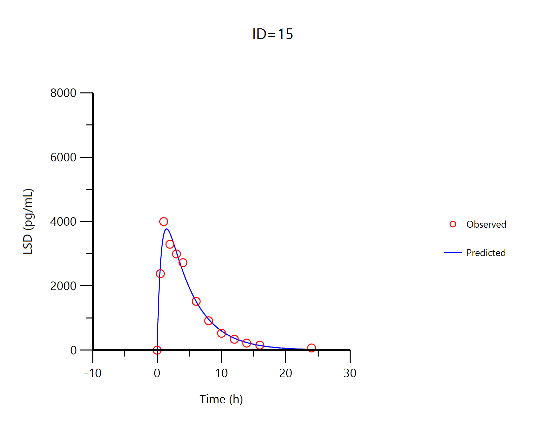

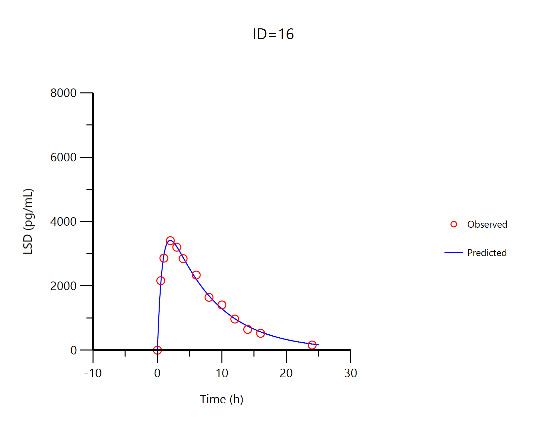

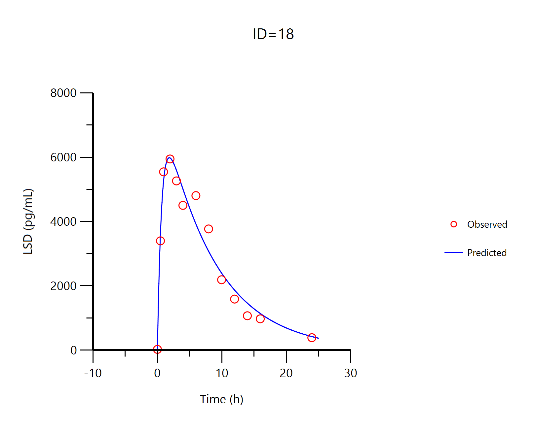

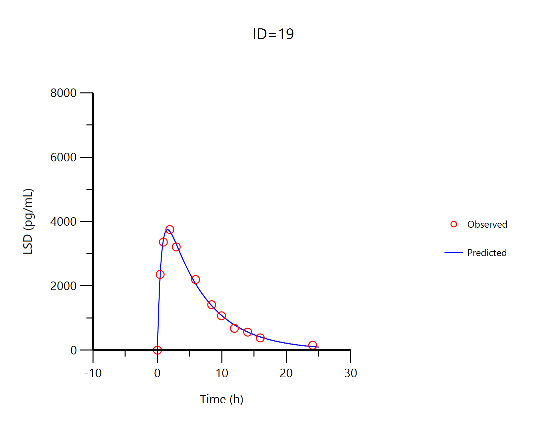

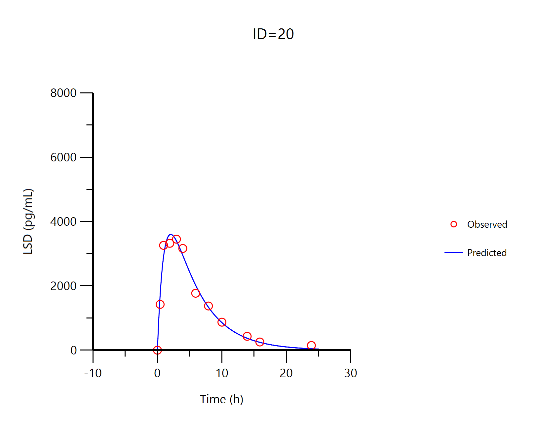


**Figure S8.** LSD plasma concentration-time curves. LSD was orally administered as a solution in ethanol at a dose of 200 µg at t = 0. The data represent individual observed LSD plasma concentrations as measured at the different time points (○) and the LSD concentrations predicted by the one-compartment pharmacokinetic model (blue lines).


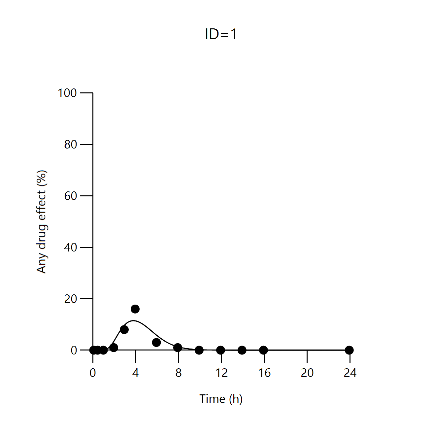

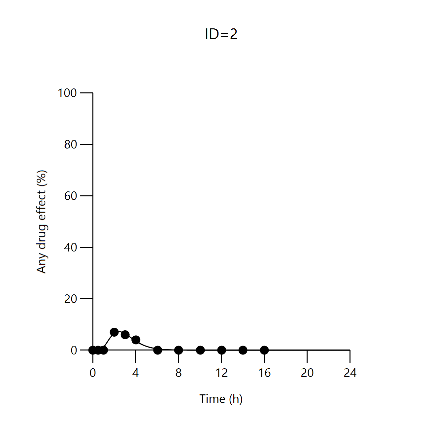

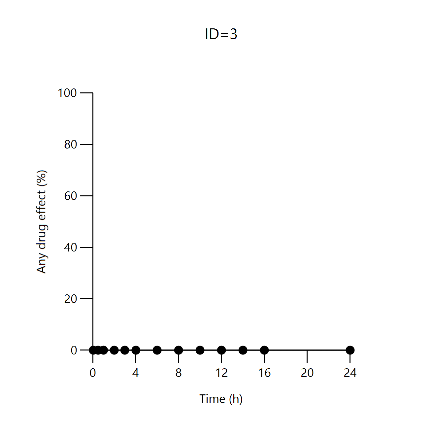

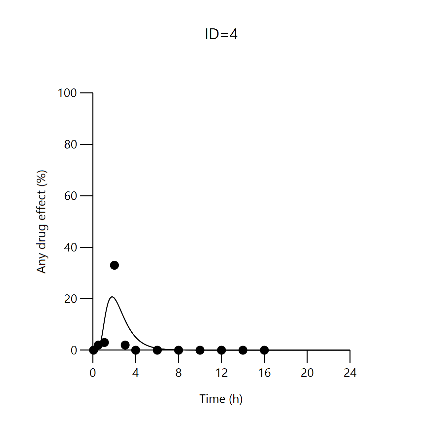

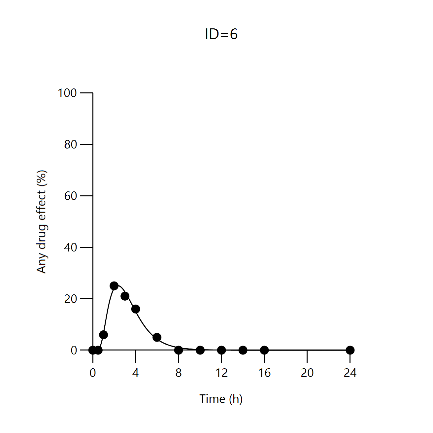

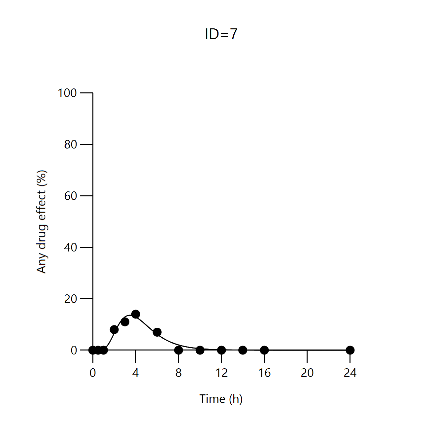

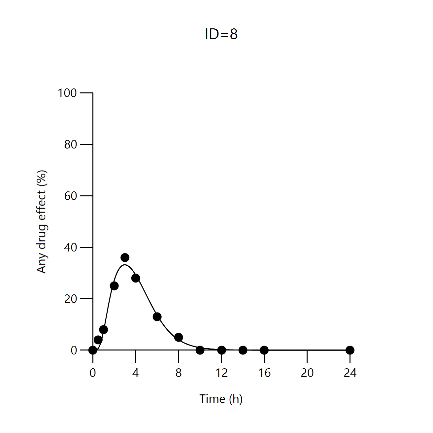

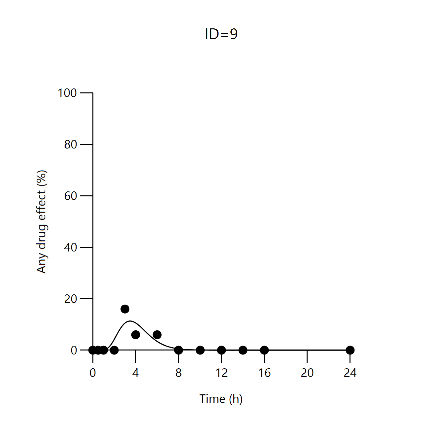

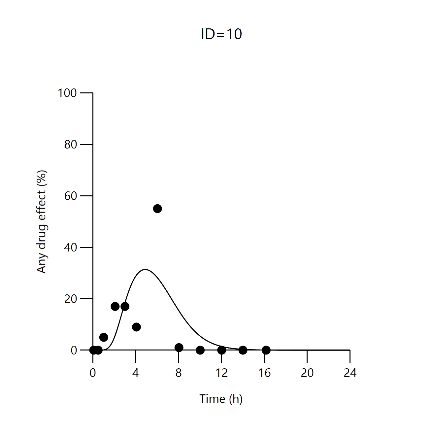

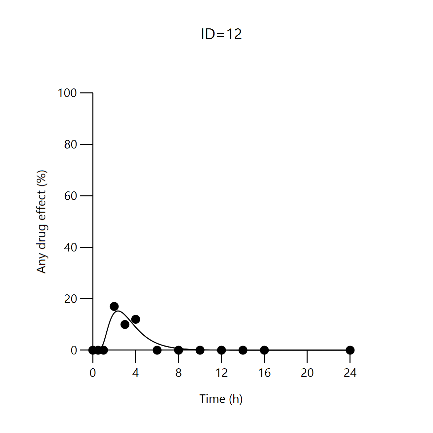

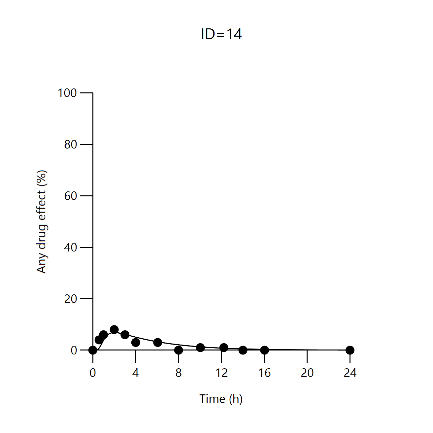

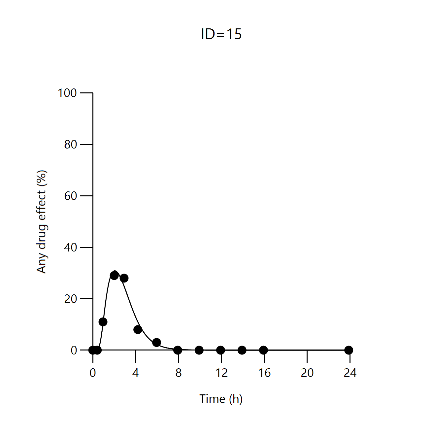

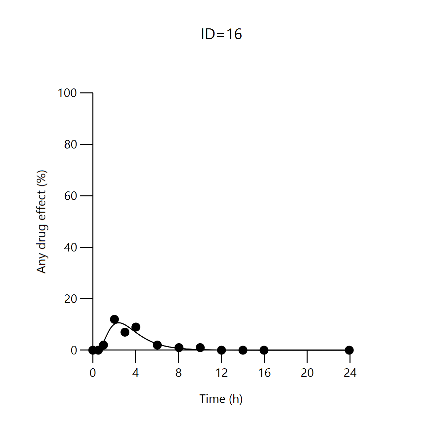

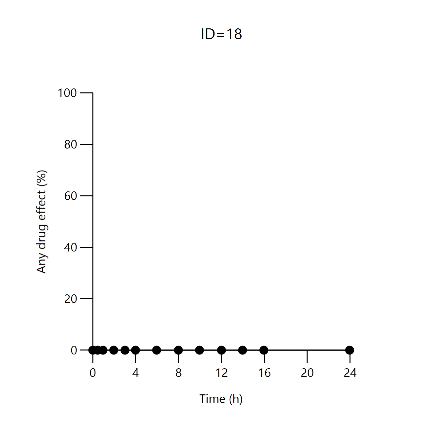

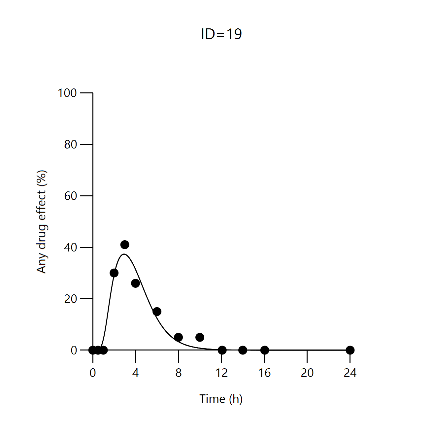

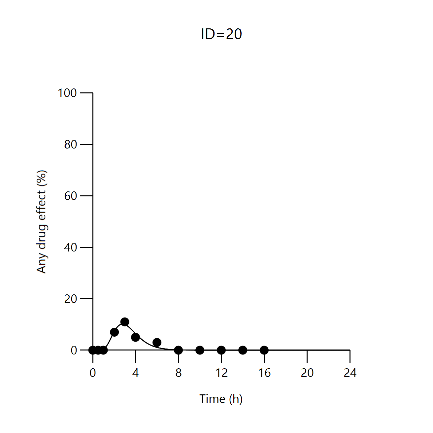


**Figure S9.** Subjective responses to LSD. LSD was orally administered as a solution in ethanol at a dose of 25 µg at t = 0 h. The data represent individual observed LSD responses on the “Any drug effect” Visual Analog Scale (rated 0-100%) at the different time points (●) and the pharmacokinetic-pharmacodynamic model-predicted effect (black lines).


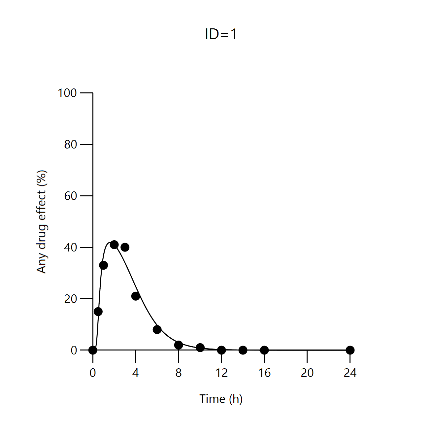

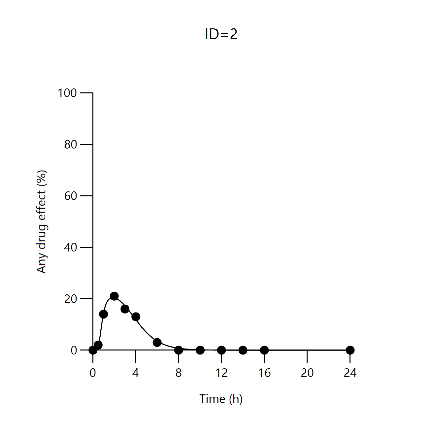

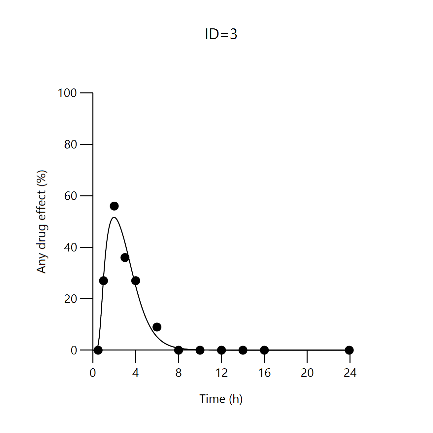

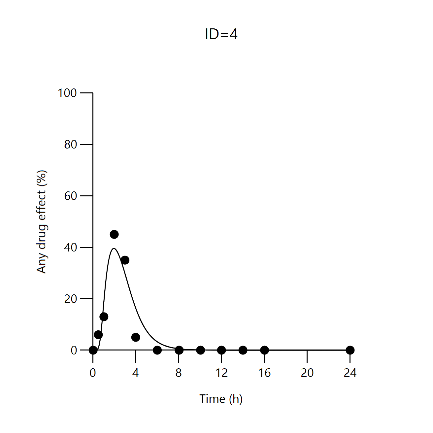

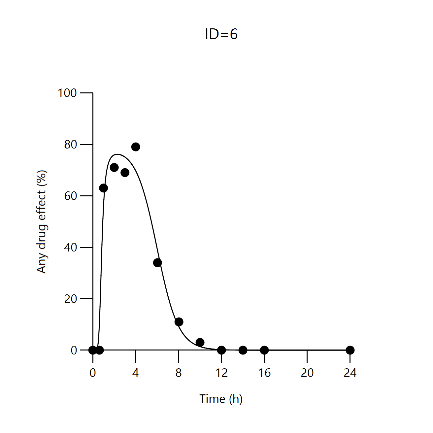

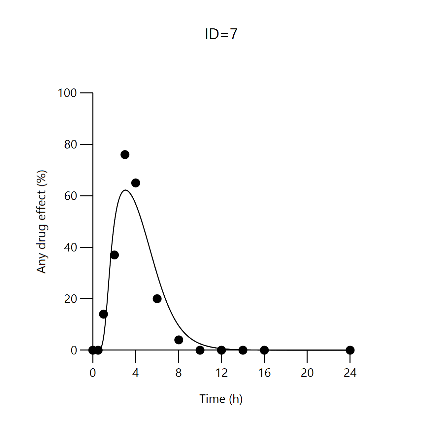

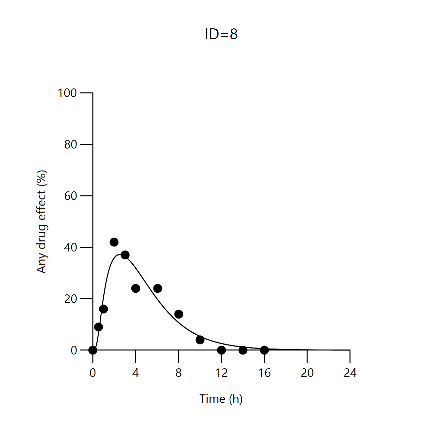

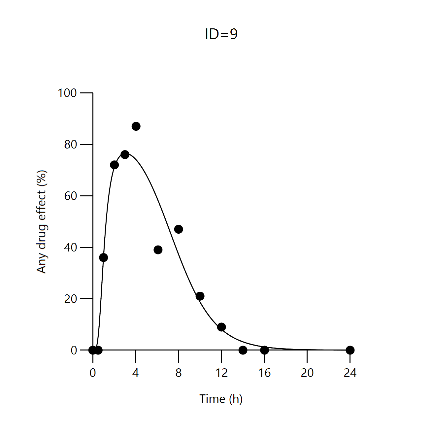

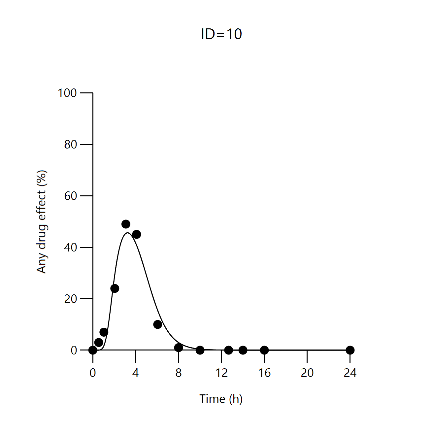

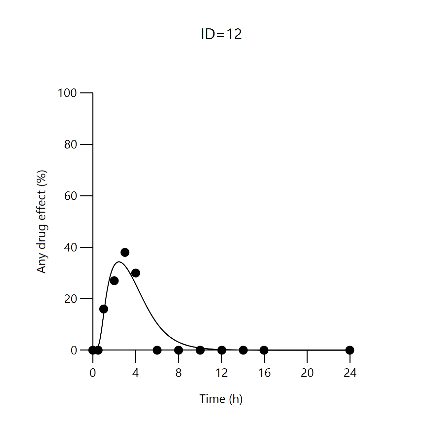

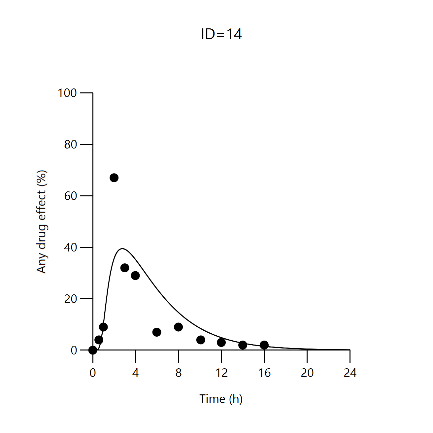

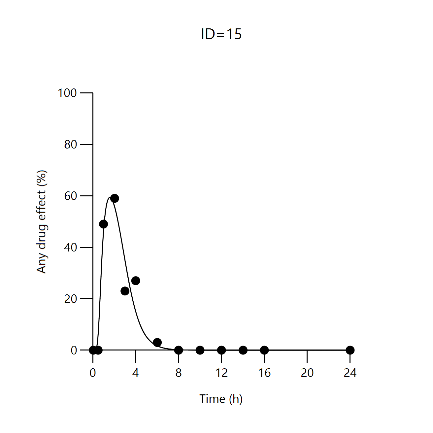

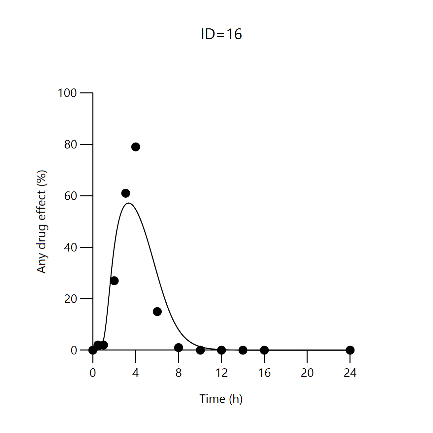

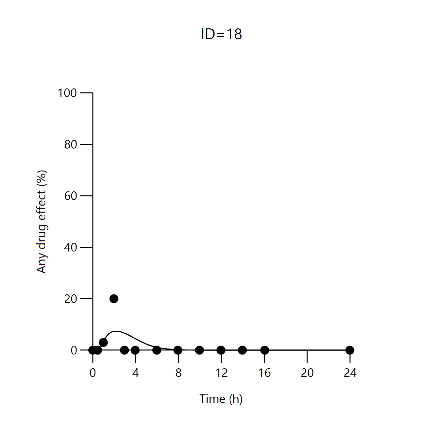

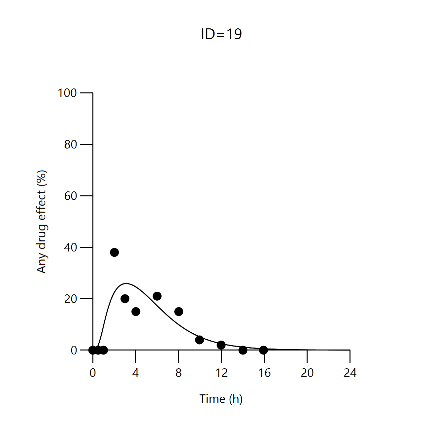

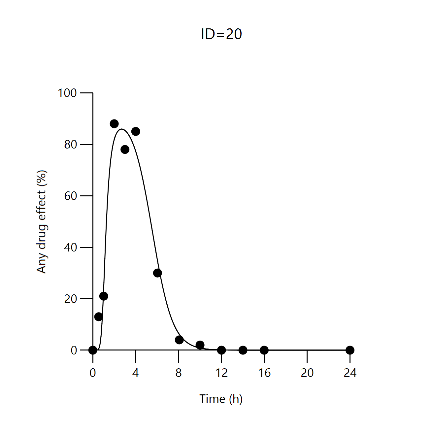


**Figure S10.** Subjective responses to LSD. LSD was orally administered as a solution in ethanol at a dose of 50 µg at t = 0 h. The data represent individual observed LSD responses on the “Any drug effect” Visual Analog Scale (rated 0-100%) at the different time points (●) and the pharmacokinetic-pharmacodynamic model-predicted effect (black lines).

**Figure S11.** Subjective responses to LSD. LSD was orally administered as a solution in ethanol at a dose of 100 µg at t = 0 h. The data represent individual observed LSD responses on the “Any drug effect” Visual Analog Scale (rated 0-100%) at the different time points (●) and the pharmacokinetic-pharmacodynamic model-predicted effect (black lines).

**Figure S12.** Subjective responses to LSD. LSD was orally administered as a solution in ethanol at a dose of 200 µg at t = 0 h. The data represent individual observed LSD responses on the “Any drug effect” Visual Analog Scale (rated 0-100%) at the different time points (●) and the pharmacokinetic-pharmacodynamic model-predicted effect (black lines).

**Figure S13.** Subjective responses to LSD. LSD was orally administered as a solution in ethanol at a dose of 25 µg at t = 0 h. The data represent individual observed LSD responses on the “Good drug effect” Visual Analog Scale (rated 0-100%) at the different time points (●) and the pharmacokinetic-pharmacodynamic model-predicted effect (black lines).

**Figure S14.** Subjective responses to LSD. LSD was orally administered as a solution in ethanol at a dose of 50 µg at t = 0 h. The data represent individual observed LSD responses on the “Good drug effect” Visual Analog Scale (rated 0-100%) at the different time points (●) and the pharmacokinetic-pharmacodynamic model-predicted effect (black lines).

**Figure S15.** Subjective responses to LSD. LSD was orally administered as a solution in ethanol at a dose of 100 µg at t = 0 h. The data represent individual observed LSD responses on the “Good drug effect” Visual Analog Scale (rated 0-100%) at the different time points (●) and the pharmacokinetic-pharmacodynamic model-predicted effect (black lines).

**Figure S16.** Subjective responses to LSD. LSD was orally administered as a solution in ethanol at a dose of 200 µg at t = 0 h. The data represent individual observed LSD responses on the “Good drug effect” Visual Analog Scale (rated 0-100%) at the different time points (●) and the pharmacokinetic-pharmacodynamic model-predicted effect (black lines).

**Figure S17.** Subjective responses to LSD. LSD was orally administered as a solution in ethanol at a dose of 25 µg at t = 0 h. The data represent individual observed LSD responses on the “Bad drug effect” Visual Analog Scale (rated 0-100%) at the different time points (●) and the pharmacokinetic-pharmacodynamic model-predicted effect (black lines).

**Figure S18.** Subjective responses to LSD. LSD was orally administered as a solution in ethanol at a dose of 50 µg at t = 0 h. The data represent individual observed LSD responses on the “Bad drug effect” Visual Analog Scale (rated 0-100%) at the different time points (●) and the pharmacokinetic-pharmacodynamic model-predicted effect (black lines).

**Figure S19.** Subjective responses to LSD. LSD was orally administered as a solution in ethanol at a dose of 100 µg at t = 0 h. The data represent individual observed LSD responses on the “Bad drug effect” Visual Analog Scale (rated 0-100%) at the different time points (●) and the pharmacokinetic-pharmacodynamic model-predicted effect (black lines).

**Figure S20.** Subjective responses to LSD. LSD was orally administered as a solution in ethanol at a dose of 200 µg at t = 0 h. The data represent individual observed LSD responses on the “Bad drug effect” Visual Analog Scale (rated 0-100%) at the different time points (●) and the pharmacokinetic-pharmacodynamic model-predicted effect (black lines).

**Figure S21.** Subjective responses to LSD. LSD was orally administered as a solution in ethanol at a dose of 25 µg at t = 0 h. The data represent individual observed LSD responses on the “Ego dissolution” Visual Analog Scale (rated 0-100%) at the different time points (●) and the pharmacokinetic-pharmacodynamic model-predicted effect (black lines).

**Figure S22.** Subjective responses to LSD. LSD was orally administered as a solution in ethanol at a dose of 50 µg at t = 0 h. The data represent individual observed LSD responses on the “Ego dissolution” Visual Analog Scale (rated 0-100%) at the different time points (●) and the pharmacokinetic-pharmacodynamic model-predicted effect (black lines).

**Figure S23.** Subjective responses to LSD. LSD was orally administered as a solution in ethanol at a dose of 100 µg at t = 0 h. The data represent individual observed LSD responses on the “Ego dissolution” Visual Analog Scale (rated 0-100%) at the different time points (●) and the pharmacokinetic-pharmacodynamic model-predicted effect (black lines).

**Figure S24.** Subjective responses to LSD. LSD was orally administered as a solution in ethanol at a dose of 100 µg at t = 0 h. The data represent individual observed LSD responses on the “Ego dissolution” Visual Analog Scale (rated 0-100%) at the different time points (●) and the pharmacokinetic-pharmacodynamic model-predicted effect (black lines).

**References**

(1) Holze, F. *et al.* Distinct acute effects of LSD, MDMA, and D-amphetamine in healthy subjects. *Neuropsychopharmacology* **45**, 462-71 (2020).

(2) Schmid, Y. *et al.* Acute effects of lysergic acid diethylamide in healthy subjects. *Biol Psychiatry* **78**, 544-53 (2015).

(3) Hysek, C.M. *et al.* MDMA enhances emotional empathy and prosocial behavior. *Soc Cogn Affect Neurosci* **9**, 1645-52 (2014).

(4) Holze, F., Duthaler, U., Vizeli, P., Muller, F., Borgwardt, S. & Liechti, M.E. Pharmacokinetics and subjective effects of a novel oral LSD formulation in healthy subjects. *Br J Clin Pharmacol* **85**, 1474-83 (2019).

(5) Dolder, P.C. *et al.* Pharmacokinetics and pharmacodynamics of lysergic acid diethylamide in healthy subjects. *Clin Pharmacokinetics* **56**, 1219-30 (2017).

(6) Tagliazucchi, E. *et al.* Increased global functional connectivity correlates with LSD-induced ego dissolution. *Curr Biol* **26**, 1043-50 (2016).

(7) Liechti, M.E., Dolder, P.C. & Schmid, Y. Alterations in conciousness and mystical-type experiences after acute LSD in humans. *Psychopharmacology* **234**, 1499-510 (2017).

(8) Janke, W. & Debus, G. *Die Eigenschaftswörterliste.* (Hogrefe: Göttingen., 1978).

(9) Dittrich, A. The standardized psychometric assessment of altered states of consciousness (ASCs) in humans. *Pharmacopsychiatry* **31 (Suppl 2)**, 80-4 (1998).

(10) Studerus, E., Gamma, A. & Vollenweider, F.X. Psychometric evaluation of the altered states of consciousness rating scale (OAV). *PLoS One* **5**, e12412 (2010).

(11) Carhart-Harris, R.L. *et al.* The paradoxical psychological effects of lysergic acid diethylamide (LSD). *Psychol Med* **46**, 1379-90 (2016).

(12) Dolder, P.C., Schmid, Y., Mueller, F., Borgwardt, S. & Liechti, M.E. LSD acutely impairs fear recognition and enhances emotional empathy and sociality. *Neuropsychopharmacology* **41**, 2638-46 (2016).

(13) Bershad, A.K., Schepers, S.T., Bremmer, M.P., Lee, R. & de Wit, H. Acute subjective and behavioral effects of microdoses of lysergic acid diethylamide in healthy human volunteers. *Biol Psychiatry* **86**, 792-800 (2019).

(14) Preller, K.H. *et al.* The fabric of meaning and subjective effects in LSD-induced states depend on serotonin 2A receptor activation *Curr Biol* **27**, 451-57 (2017).

(15) Roseman, L., Nutt, D.J. & Carhart-Harris, R.L. Quality of acute psychedelic experience predicts therapeutic efficacy of psilocybin for treatment-resistant depression. *Front Pharmacol* **8**, 974 (2017).

(16) Griffiths, R.R. *et al.* Psilocybin produces substantial and sustained decreases in depression and anxiety in patients with life-threatening cancer: a randomized double-blind trial. *J Psychopharmacol* **30**, 1181-97 (2016).

(17) Griffiths, R.R., Richards, W.A., McCann, U. & Jesse, R. Psilocybin can occasion mystical-type experiences having substantial and sustained personal meaning and spiritual significance. *Psychopharmacology (Berl)* **187**, 268-83; discussion 84-92 (2006).

(18) Barrett, F.S., Johnson, M.W. & Griffiths, R.R. Validation of the revised Mystical Experience Questionnaire in experimental sessions with psilocybin. *J Psychopharmacol* **29**, 1182-90 (2015).

(19) MacLean, K.A., Johnson, M.W. & Griffiths, R.R. Mystical experiences occasioned by the hallucinogen psilocybin lead to increases in the personality domain of openness. *J Psychopharmacol* **25**, 1453-61 (2011).

(20) Griffiths, R.R., Johnson, M.W., Richards, W.A., Richards, B.D., McCann, U. & Jesse, R. Psilocybin occasioned mystical-type experiences: immediate and persisting dose-related effects. *Psychopharmacology (Berl)* **218**, 649-65 (2011).

(21) Griffiths, R., Richards, W., Johnson, M., McCann, U. & Jesse, R. Mystical-type experiences occasioned by psilocybin mediate the attribution of personal meaning and spiritual significance 14 months later. *J Psychopharmacol* **22**, 621-32 (2008).

(22) Garcia-Romeu, A., Griffiths, R.R. & Johnson, M.W. Psilocybin-occasioned mystical experiences in the treatment of tobacco addiction. *Curr Drug Abuse Rev* **7**, 157-64 (2015).

(23) Garcia-Romeu, A., Davis, A.K., Erowid, F., Erowid, E., Griffiths, R.R. & Johnson, M.W. Cessation and reduction in alcohol consumption and misuse after psychedelic use. *J Psychopharmacol*, 269881119845793 (2019).

(24) Griffiths, R.R. *et al.* Psilocybin-occasioned mystical-type experience in combination with meditation and other spiritual practices produces enduring positive changes in psychological functioning and in trait measures of prosocial attitudes and behaviors. *J Psychopharmacol* **32**, 49-69 (2018).

(25) Ross, S. *et al.* Rapid and sustained symptom reduction following psilocybin treatment for anxiety and depression in patients with life-threatening cancer: a randomized controlled trial. *J Psychopharmacol* **30**, 1165-80 (2016).
