# Supplementary material for: Acute dose-dependent effects of lysergic acid diethylamide in a double-blind placebo-controlled study in healthy subjects
Source: Neuropsychopharmacology. 2020 Oct 15;46(3):537–44. doi: 10.1038/s41386-020-00883-6 (PMC8027607; doi:10.1038/s41386-020-00883-6)
Supplement: Supplementary file 10 — Consort Flow Chart [file 41386_2020_883_MOESM10_ESM.pptx]

## Slide 1
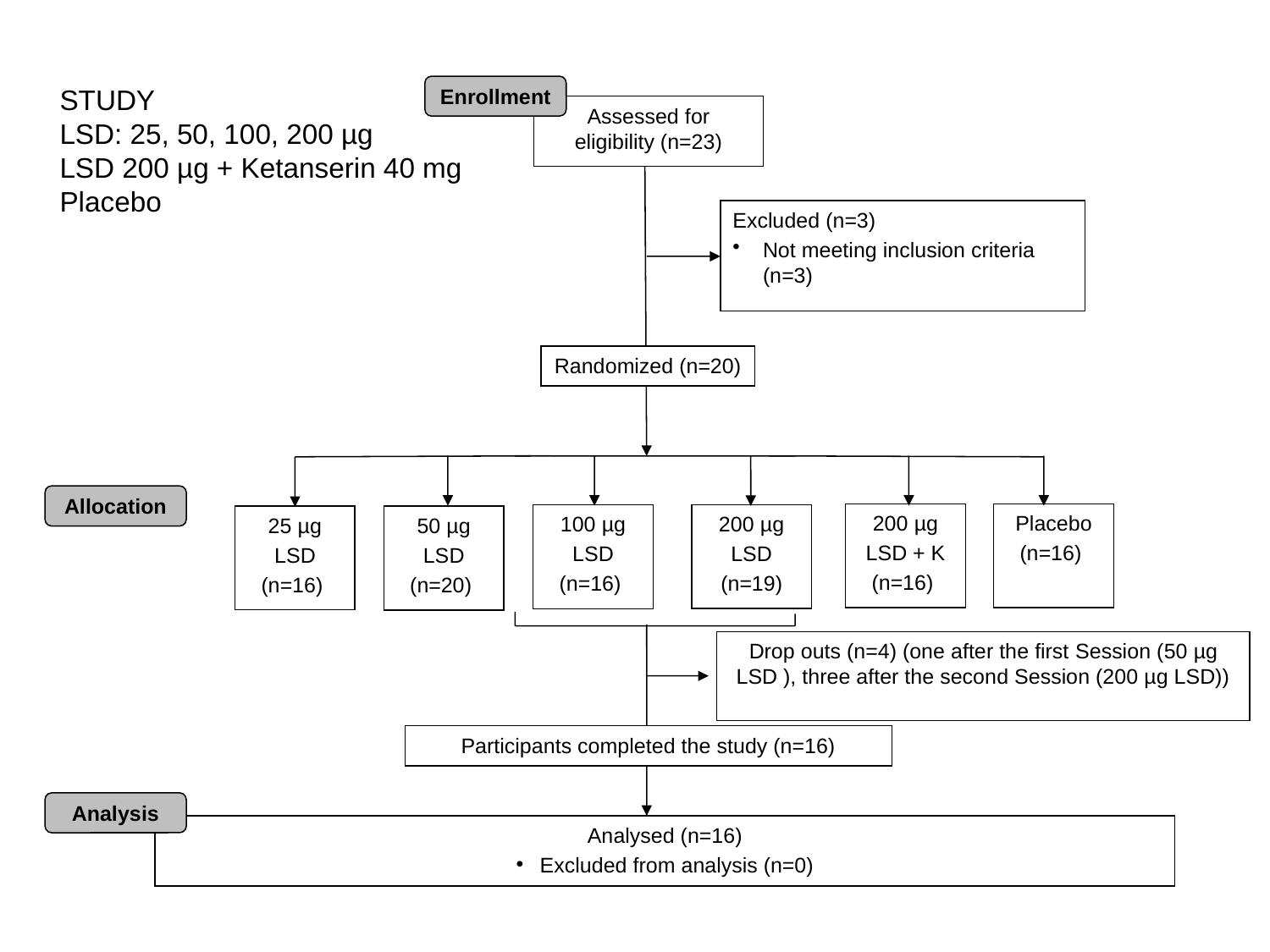

STUDY
LSD: 25, 50, 100, 200 µg
LSD 200 µg + Ketanserin 40 mg
Placebo
Enrollment
Assessed for eligibility (n=23)
Excluded (n=3)
Not meeting inclusion criteria (n=3)
Randomized (n=20)
Allocation
200 µg
LSD + K
(n=16)
Placebo
(n=16)
100 µg
LSD
(n=16)
200 µg
LSD
(n=19)
25 µg
LSD
(n=16)
50 µg
LSD
(n=20)
Drop outs (n=4) (one after the first Session (50 µg LSD ), three after the second Session (200 µg LSD))
Participants completed the study (n=16)
Analysis
Analysed (n=16)
Excluded from analysis (n=0)
